# Adaptive spline-based weighting functions for blended survival curve extrapolation

**DOI:** 10.1177/09622802261458072

**Published:** 2026-07-16

**Authors:** Hao Chen, Clara Grazian

**Affiliations:** 1 School of Mathematics and Statistics, 4334 The University of Sydney, Sydney, NSW, Australia; 2 ARC Training Centre in Data Analytics for Resources and Environments (DARE), NSW, Australia

**Keywords:** Survival extrapolation, blended survival curves, monotone P-splines, shape-constrained additive models, Gompertz tail, survival anchor, health technology assessment

## Abstract

Health technology assessment frequently requires survival predictions well beyond the observed trial follow-up, yet single parametric models fitted to short horizons can accumulate long-term bias. Current guidance also encourages the principled use of external evidence, such as registry summaries or expert anchors, while maintaining fidelity to the trial data. We introduce an adaptive spline-weighted blended extrapolation on the cumulative-hazard scale that unifies these aims. The observation side is fitted on the trial window using a piecewise-exponential Cox-type model with a smooth prior-driven continuation beyond the administrative cutoff, implemented via INLA. The external side is an anchored Gompertz tail identified by a prespecified survival level at a clinically relevant time. To blend the two components, we compare their cumulative hazards, learn a monotone P-spline score over time, and pass it through a logistic link to obtain a data-driven, time-varying weight. A simple “temperature” scaling controls the slope of this weight and provides a practical guarantee of non-negative blended hazards on a chosen grid, preserving adaptivity while ensuring feasibility. Across Monte Carlo scenarios spanning multiple tail shapes and censoring levels, the method delivers consistently lower absolute survival error, smaller restricted mean survival time error, and improved stability compared with fixed-schedule blending and single-family parametric models. In a SEER registry study with three-year observation and ten-year extrapolation, the blended curve tracks the Kaplan–Meier estimates within follow-up and transitions smoothly toward the anchored tail, yielding small long-horizon errors across cancer sites and age strata. The framework is modular, interpretable, and easily extended to alternative tails and multiple anchors. An open-source implementation is available in the R package survblendr at https://github.com/haohaostats/survblendr.

## 1. Introduction

Survival analysis is the most common statistical framework for evaluating time-to-event outcomes, with classical methods including the Kaplan–Meier non-parametric estimator^1^ and the Cox proportional hazards model.^2^ However, in health technology assessment (HTA) and long-term follow-up studies such as in oncology, the available trial follow-up often covers only a limited portion of the period relevant for decision-making, while clinicians and policy-makers typically need survival estimates over a much longer horizon.^3^ This creates a familiar practical difficulty: the available trial data may describe short-term or medium-term outcomes reasonably well, but reimbursement and policy decisions often require survival projections far beyond the observed follow-up window. A major challenge is that the hazard function over a full decision horizon may change shape in ways that are difficult to represent well with a single global parametric family. In oncology, for example, short-term survival may reflect an initial treatment effect, later survival may be influenced by disease progression or depletion of higher-risk patients, and still later outcomes may be increasingly shaped by background mortality. In such settings, one model may fit the observed portion of follow-up adequately while still implying an implausible long-term tail. Directly fitting a single parametric family across the entire timeline can therefore lead to the accumulation of long-term bias due to model misspecification.^3^ To mitigate this issue, considerable work has proposed more flexible modeling and piecewise extrapolation on the log-cumulative hazard or log-survival scale. Examples include log-cumulative hazard regression based on Royston–Parmar flexible parametric models,^4–6^ and the implementation of generalized parametric survival families.^7^

At the level of evidence synthesis, HTA guidelines recommend systematically incorporating external evidence or external information into long-term extrapolation while ensuring a good short-term fit.^3^ Such inputs may come from registry data, historical cohorts, published summaries, or clinically elicited long-term survival expectations. A natural strategy is therefore to combine two complementary components: an observation-side curve, fitted primarily to the trial data up to $t_{\mathrm{obs}}$ , and an external-side curve, reflecting longer-term survival informed by sources beyond the trial. Blended survival models provide one way to formalize this idea, allowing the extrapolated curve to remain close to the trial evidence early on while gradually incorporating externally informed long-term behavior later.^8^ Recent work on blended survival curves^8^ has shown that this strategy can improve long-term plausibility relative to relying on a single parametric model alone. However, existing fixed-schedule approaches typically specify in advance how the transition from the observation-side curve to the external-side curve should occur over time, for example through a Beta cumulative distribution function over a pre-defined window. This can be restrictive when the discrepancy between the two curves is not uniform across the transition period. For example, the two curves may remain close immediately after the end of follow-up and then separate more sharply later, or they may diverge soon after the cutoff and then remain stably apart. In such situations, a pre-specified transition schedule may shift weight to the external curve too early or too late. The resulting extrapolation may still be smooth, but the timing and pace of the transition may not be well matched to the actual disagreement between the short-term and long-term sources.^3,7^

Motivated by this limitation, this paper proposes an adaptive spline-weighted blended extrapolation framework with three complementary aims: (i) to preserve consistency with the observed trial evidence and its smooth continuation beyond the end of follow-up; (ii) to incorporate anchored external information about longer-term survival into the extrapolated tail; and (iii) to allow the transition between the two components to respond adaptively to their evolving discrepancy over time. We first construct an observation-side survival curve that captures the observed trial pattern and allows a smooth continuation beyond the administrative cutoff. We then specify an external long-term tail using an anchored parametric form that incorporates external survival information at a clinically relevant future time point. Finally, instead of imposing a fixed transition schedule, we estimate a time-varying weight that adapts to the contrast between these two components on the cumulative-hazard scale. For the observation-side component, we use a flexible piecewise exponential formulation related to the Cox model, together with a smooth prior-driven continuation beyond the observed follow-up period; computation is carried out using INLA.^9–11^ For the external component, we use an anchor-identifiable Gompertz tail.^12^ The adaptive transition is constructed using a monotone P-spline fitted on the cumulative-hazard contrast scale, with monotonicity enforced through a Shape-Constrained Additive Model (SCAM) and P-spline penalization.^13,14^ A key technical challenge in such adaptive blending is ensuring hazard non-negativity. While linear blending on the cumulative-hazard scale is interpretable, the implied hazard includes an additional drift term induced by changes in the weight over time. To address this, we introduce a simple temperature scaling parameter that controls how rapidly the adaptive weight is allowed to vary, and we use it to provide a practical guarantee of nonnegative blended hazards on the computational grid. For evaluation, in addition to conventional pointwise absolute error, we use the restricted mean survival time (RMST)^15,16^ as a long-term integrated error metric. RMST avoids reliance on the proportional hazards assumption and is widely used for trial comparison and extrapolation calibration.^7,15,16^ In our real-data application, we use several large-scale cohorts from the SEER cancer registry to conduct 3-year observation and 10-year extrapolation simulated censoring experiments, testing the method’s robustness across different cancer types and age groups.^17^

In summary, the contributions of this paper are threefold: **(i)** we propose an adaptive blending framework on the cumulative hazard scale that unifies smooth continuation of the observation-side model with an anchor-identifiable external tail; **(ii)** through a monotone P-spline combined with temperature scaling, we provide an operational guarantee for hazard non-negativity; **(iii)** using both simulations and real SEER data, we systematically compare the long-term extrapolation stability and accuracy of our adaptive blending approach against fixed-schedule and single-family parametric models, presenting results with multiple metrics including RMST and pointwise errors. Our empirical results demonstrate that the adaptive weights can effectively capture time-varying discrepancies and maintain robust extrapolation performance across a wide range of tail shapes.^3,4,7,8^ The remainder of the paper is organized as follows. Section 2 formalizes the cumulative-hazard blending framework, details the observation-side PEM/INLA fit, the anchor-identified Gompertz tail, and the adaptive monotone P-spline weighting with temperature control, and summarizes the estimation algorithm. Section 3 designs Monte Carlo experiments and reports comparative results against baselines using survival, RMST, and stability-based metrics. Section 4 applies the method to SEER registry cohorts under a three-year observation and ten-year extrapolation setting, illustrating practical performance across cancer sites and age strata. Section 5 concludes and discusses limitations and extensions.

## 2. Methods

Survival analysis concerns a non-negative time-to-event variable T , with survival function S(t)=Pr(T>t) , cumulative hazard H(t)=−logS(t) , and (instantaneous) hazard $h(t)=H^{\prime }(t)$ for continuous T . Let $t_{\mathrm{obs}}$ denote the end of the observed follow-up period and let $T_{⋆}>t_{\mathrm{obs}}$ denote the extrapolation horizon of interest. In many clinical trials, follow-up ends at $t_{\mathrm{obs}}$ , whereas in HTA such data are often used to inform decisions over the longer horizon $\left[t_{\mathrm{obs}},T_{⋆}\right]$ . Extrapolation is therefore unavoidable and often benefits from judicious use of external evidence, including external data sources and externally elicited long-term survival information. We develop a blending approach that combines (i) short-term evidence from the trial, modeled flexibly on $\left[0,t_{\mathrm{obs}}\right]$ , and (ii) longer-term structure represented through an anchored parametric tail. The central design choice is the time-varying weight π(t)∈[0,1] that governs the transition. We first formalize the setting and the blending identity, then review a fixed-schedule Beta weighting as a comparator, and finally present our adaptive spline-blended method with identifiability and validity guarantees, together with an estimation algorithm and implementation details.

### 2.1. Set-up, notation, and goals

Let T≥0 denote the event time. We observe right-censored outcomes on $\left[0,t_{\mathrm{obs}}\right]$ , and wish to extrapolate up to an extrapolation horizon $T_{⋆}>t_{\mathrm{obs}}$ . Here $C_{i}$ denotes the (potential) right-censoring time for subject i (e.g. loss to followup or withdrawal), distinct from the administrative cutoff $t_{\mathrm{obs}}$ . Equivalently, for subjects i=1,…,n , let $T_{i}$ denote the (true) event time and $C_{i}$ the potential right-censoring time; define ${\tilde{C}}_{i}=\min(C_{i},t_{\mathrm{obs}})$ and write $Y_{i}=\min(T_{i},{\tilde{C}}_{i})$ and $\Delta_{i}=1_{\left\{T_{i}\leq {\tilde{C}}_{i}\right\}}$ . Here $Y_{i}$ is the observed (follow-up) time, and $x_{i}$ denotes optional covariates. We assume non-informative right-censoring on $\left[0,t_{\mathrm{obs}}\right]$ . Two time-indexed components are defined on the full horizon $\left[0,T_{⋆}\right]$ . The first is the observation-side component, represented by $S_{\mathrm{obs}}(t)$ , $H_{\mathrm{obs}}(t)$ , and $h_{\mathrm{obs}}(t)$ for $t\in [0,T_{⋆}]$ . This component is fitted using only data observed up to $t_{\mathrm{obs}}$ , and is then smoothly extended beyond the observation window; the modeling details are given in Section 2.4.1. The second is the external component, represented by $S_{\mathrm{ext}}(t)$ , $H_{\mathrm{ext}}(t)$ , and $h_{\mathrm{ext}}(t)$ for $t\in [0,T_{⋆}]$ . This component is parameterized to reflect longer-term survival using external evidence or externally elicited information, and is identified by one or more anchors beyond $t_{\mathrm{obs}}$ (Section 2.4.2). Although we refer to it as the “tail” model because its identification comes from anchor specifications beyond $t_{\mathrm{obs}}$ , it is algebraically defined on the entire interval $\left[0,T_{⋆}\right]$ . External inputs are summarized at anchor times $t_{a}\in [t_{\mathrm{obs}},T_{⋆}]$ as survival levels $S_{a}\in (0,1)$ that identify the external parameterization. These anchors are not treated as observed outcome data in model fitting; rather, they are treated as prior information used to identify the external tail. Numerical evaluations use a grid $\left\{t_{g}{\}}_{g=1}^{G}\subset [0,T_{⋆}\right]$ .

Our goal is to extrapolate S(t) over $\left[t_{\mathrm{obs}},T_{⋆}\right]$ by blending the two full-horizon components through a time-varying weight π(t)∈[0,1] ; modeling details follow in subsequent subsections (the anchor specification and its uncertainty are given in Section 2.4.2). In words, $S_{\mathrm{obs}}(t)$ is the data-driven curve, with a smooth continuation beyond the observation window; $S_{\mathrm{ext}}(t)$ is the externally anchored curve; and π(t) blends them over time. Unless otherwise stated, accuracy metrics are computed on the extrapolation window $\left[t_{\mathrm{obs}},T_{⋆}\right]$ while figures may display curves on $\left[0,T_{⋆}\right]$ for context. Symbols used below are collected in Table 1.

**Table 1.** Notation used in methods.

Symbol	Meaning
$t_{\mathrm{obs}}$	end of the observed follow-up period
$T_{⋆}$	extrapolation horizon $\left(>t_{\mathrm{obs}}\right)$
$t_{a}$	anchor time(s) for external evidence or externally elicited information
$T_{i}$	true event time (possibly unobserved)
$x_{i}$	covariates (optional)
$S_{a}$	anchor survival level at time $t_{a}$ , treated as prior information
$1_{A}$	indicator function of event A
n	sample size
J	number of monotone P-spline basis functions
G	number of time grid points for numerical evaluation
$S_{\mathrm{obs}},H_{\mathrm{obs}},h_{\mathrm{obs}}$	survival/cum. hazard/hazard functions from the observation-side model
$S_{\mathrm{ext}},H_{\mathrm{ext}},h_{\mathrm{ext}}$	survival/cum. hazard/hazard functions from the external tail model
$S_{\mathrm{blend}},H_{\mathrm{blend}},h_{\mathrm{blend}}$	blended survival/cum. hazard/hazard functions
$\theta_{\mathrm{obs}}$	parameters of the observation-side model
$\theta_{\mathrm{ext}}=(\gamma ,S_{a})$	parameters of the external Gompertz tail (with $\rho =\rho (\gamma ,S_{a})$ )
z(t)	log-cum. hazard contrast, i.e. $\log\;H_{\mathrm{obs}}(t)-\log\;H_{\mathrm{ext}}(t)$
η(t)	monotone latent score fitted to z(t)
π(t)	time-varying blending weight defined in [0,1]
$B_{j}(t)$	j -th monotone-increasing P-spline basis (SCAM “mpi”)
$\xi =(\xi_{1},\ldots ,\xi_{J})$	nonnegative spline coefficients in $\eta (t)=\sum_{j}\xi_{j}B_{j}(t)$
τ	temperature for slope control (hazard nonnegativity)
$C_{i}$	individual right-censoring time (loss to follow-up/withdrawal)
${\tilde{C}}_{i}=\min(C_{i},t_{\mathrm{obs}})$	effective censoring time within the observation window
$Y_{i},\Delta_{i}$	observed time and event indicator ( $Y_{i}=\min(T_{i},{\tilde{C}}_{i})$ , $\Delta_{i}=1_{\left\{T_{i}\leq {\tilde{C}}_{i}\right\}}$ )
$\{t_{g}{\}}_{g=1}^{G}$	time grid for numerical evaluation on $\left[0,T_{⋆}\right]$

Figure 1 provides a graphical summary of the extrapolation problem and the logic of our proposed method through a concrete illustrative example. Suppose we have a clinical trial with a follow-up period of 10 years ( $t_{\mathrm{obs}}=10$ ), while the quantity of interest is survival at 20 years ( $T_{⋆}=20$ ). In this example, the interval from 10 to 20 years is the extrapolation window: patient-level survival outcomes are observed only over the first 10 years, but survival must still be projected beyond the end of trial follow-up. We also suppose that an external source provides a 20-year survival anchor, $S_{a}=0.25$ , shown by the red point in all three panels. Such information may come, for example, from clinicians’ experience with similar patient populations, historical registry summaries, published long-term follow-up studies, or other external survival benchmarks available beyond the trial window.

**Figure 1.**
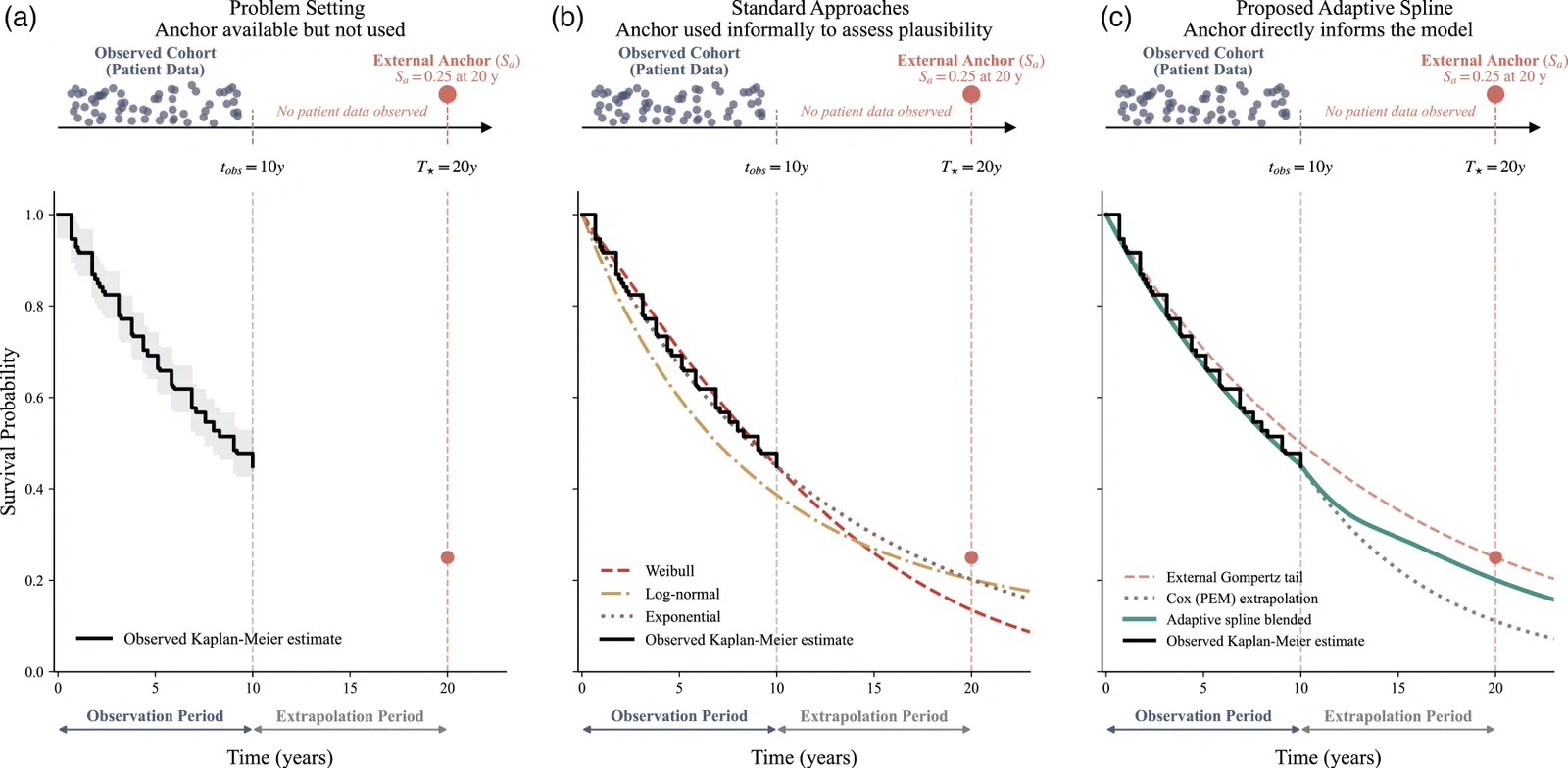
Conceptual overview of the extrapolation setting and the proposed adaptive spline-blended approach. In the illustrative example, the external anchor is a prespecified 20-year survival level, $S_{a}=0.25$ , shown by the red point in all three panels. Panel (a) illustrates the basic extrapolation setting: survival data are observed only up to the follow-up cutoff $t_{\mathrm{obs}}$ , while extrapolation is required over a longer horizon; the anchor is available but is not used in the displayed non-parametric Kaplan–Meier estimate. Panel (b) shows standard extrapolation based on single-family parametric models, where the external anchor may be used informally to assess long-term plausibility or to help identify the least implausible parametric extrapolation. Panel (c) illustrates the proposed framework, in which an observation-side component fitted to the observed data is combined with an externally anchored tail through an adaptive spline-based transition, so that the anchor directly informs the long-term extrapolated component and the blended estimate.

Panel (A) shows the basic extrapolation setting. The external anchor is available, but it is not used in the displayed non-parametric Kaplan–Meier estimate, which is based only on the observed patient-level data up to $t_{\mathrm{obs}}$ . Panel (B) illustrates a common strategy based on fitting standard single-family parametric models. In practice, the external anchor may still be used informally to assess long-term plausibility or to help identify the least implausible parametric extrapolation, even though it does not directly enter the parametric model fit shown in the panel. These models can therefore produce markedly different long-term projections despite fitting the observed 10-year window similarly well. Panel (c) summarizes our proposed approach, in which the anchor directly informs the externally anchored Gompertz tail and, through the adaptive spline-based weight, the final blended extrapolation. The observation-side component captures the observed trial data, the external anchor defines a clinically interpretable long-term reference tail, and the adaptive spline-based weight determines how the extrapolated survival estimate transitions between trial evidence and the external target. The figure is intended as a conceptual guide; the formal model specification is given in the subsections that follow.

### 2.2. Blended survival on the cumulative-hazard scale

We blend on the log-survival (equivalently cumulative-hazard) scale:

(1) $$\log\;S_{\mathrm{blend}}(t)=(1-\pi (t))\log\;S_{\mathrm{obs}}(t)+\pi (t)\log\;S_{\mathrm{ext}}(t),$$

or

(2) $$H_{\mathrm{blend}}(t)=(1-\pi (t))H_{\mathrm{obs}}(t)+\pi (t)H_{\mathrm{ext}}(t).$$

Equivalently, $S_{\mathrm{blend}}(t)=S_{\mathrm{obs}}{\left(t\right)}^{\;1-\pi (t)}S_{\mathrm{ext}}{\left(t\right)}^{\;\pi (t)}$ (a geometric mixture); see Proposition A.1 in Appendix A for the equivalence of (1) and (2). Here π(t)∈[0,1] is a time-varying weight that typically increases over t , transferring influence from $S_{\mathrm{obs}}(t)$ to $S_{\mathrm{ext}}(t)$ . Differentiation yields a continuous blended hazard

(3) $$h_{\mathrm{blend}}(t)=(1-\pi (t))\;h_{\mathrm{obs}}(t)+\pi (t)\;h_{\mathrm{ext}}(t)+\{H_{\mathrm{ext}}(t)-H_{\mathrm{obs}}(t)\}\pi^{\prime }(t).$$

Equation (3) follows by differentiating (2) and applying the product rule; see Proposition A.2 in Appendix A. Here $S_{\mathrm{obs}}(t),H_{\mathrm{obs}}(t),h_{\mathrm{obs}}(t)$ denote the survival, cumulative hazard, and hazard functions from the observation-side model; $S_{\mathrm{ext}}(t),H_{\mathrm{ext}}(t),h_{\mathrm{ext}}(t)$ are the corresponding quantities from the external-tail model; and $S_{\mathrm{blend}}(t),H_{\mathrm{blend}}(t),h_{\mathrm{blend}}(t)$ are the blended counterparts defined by (1)–(3).

### 2.3. Fixed-schedule Beta weight

The fixed-schedule design introduced by Che et al.^8^ specifies π(t) via the Beta CDF:

(4) $$\pi_{\beta }(t;\alpha ,\beta ,a,b)=F_{\mathrm{Beta}}\left(\frac{t-a}{b-a}\;|\;\alpha ,\beta \right),\;t\in [a,b],$$

with $\pi_{\beta }(t)=0$ for t≤a and $\pi_{\beta }(t)=1$ for t≥b . The window [a,b] (often $a=t_{\mathrm{obs}},\;b=T_{⋆}$ ) controls the transition range, while α,β>0 shape the curvature (e.g. α=β=3 yields an S-curve). By the chain rule, $\pi_{\beta }^{\prime }(t)=f_{\mathrm{Beta}}((t-a)/(b-a)|\alpha ,\beta )/(b-a)$ , so the drift term in (3) scales with both the Beta density and the window width (b−a) . Hence the blended curve coincides with $S_{\mathrm{obs}}(t)$ on $\left[0,t_{\mathrm{obs}}\right]$ and with $S_{\mathrm{ext}}(t)$ at $T_{⋆}$ . This parametric schedule ensures continuity, interpretability, and ease of implementation; it also enforces $S_{\mathrm{blend}}(a)=S_{\mathrm{obs}}(a)$ and $S_{\mathrm{blend}}(b)=S_{\mathrm{ext}}(b)$ . However, its rigidity can misrepresent complex time-varying divergences between $H_{\mathrm{obs}}(t)$ and $H_{\mathrm{ext}}(t)$ , particularly when long-term hazards bend or inflect in ways inconsistent with a fixed-shape schedule. By “fixed-schedule” we mean that once (a,b,α,β) are set, the entire time course of $\pi_{\beta }(t)$ is predetermined and does not adapt to the time-varying contrast between $H_{\mathrm{obs}}(t)$ and $H_{\mathrm{ext}}(t)$ ; in particular, when α,β>1 , the fastest transition occurs at $t^{⋆}=a+(b-a)\frac{\alpha -1}{\alpha +\beta -2}$ . Practically, (α,β) control the early/late steepness (with α=β giving a symmetric S-curve), while (a,b) control the transition window and thus the maximal slope of π(t) ; narrower (b−a) induces larger $\pi^{\prime }(t)$ and a stronger drift in (3).

### 2.4. Our proposed method: Adaptive spline-blended extrapolation

We replace the fixed schedule by a data-driven monotone weight estimated from the relative evidence in the short-term fit versus the external tail. The construction is modular: a flexible observation-side model on $\left[0,t_{\mathrm{obs}}\right]$ , an identifiable external tail on $\left[t_{\mathrm{obs}},T_{⋆}\right]$ built from anchors, and an adaptive monotone P-spline mapping their contrast into π(t) .

#### 2.4.1. Observation-side model on $\left[0,t_{\text{obs}}\right]$

As noted in Section 2.1, the observation-side component is fitted using only data observed up to $t_{\mathrm{obs}}$ and then smoothly extended beyond the observation window. To do this, we use a piecewise exponential model as a flexible approximation to a Cox-type model.^9^ Let $0=t_{0}<t_{1}<\cdots <t_{K}=t_{\mathrm{obs}}$ partition the observation window $\left[0,t_{\mathrm{obs}}\right]$ . Within interval k , the baseline hazard $h_{0}(t)=h_{0k}$ is constant and the counting process likelihood reduces to Poisson regression with exposure offsets. For subject i and interval k ,

(5) $$\begin{array}{rl} y_{ik} & ∼\mathrm{Poisson}(\mu_{ik}), \end{array}$$

$$\begin{array}{rl} \mu_{ik} & =E_{ik}\;h_{0k}\exp\;(x_{i}^{⊤}\beta ). \end{array}$$

Here $y_{ik}\in \{0,1\}$ indicates whether subject i experiences the event in interval k (at most one event overall), and $E_{ik}$ is the at-risk exposure (person-time) of subject i within interval k ; the log-exposure $\log\;E_{ik}$ enters as an offset in the Poisson regression. To induce smoothness and clinical plausibility, we place a first-order random-walk (RW1) prior on the log-baseline,

(6) $$\log\;h_{0k}=\log\;h_{0,k-1}+\varepsilon_{k},\;\varepsilon_{k}|\tau_{h}∼N(0,\tau_{h}^{-1}),k=2,\ldots ,K.$$

A weakly informative prior is assigned to the RW1 precision via the log-Gamma specification,

$$\log\;\tau_{h}∼\mathrm{LogGamma}(0.01,\;0.01).$$

We use the variance-scaled version of the RW1 so that the marginal prior on the latent baseline is approximately invariant to the time partition.^18,19^ We do not impose a sum-to-zero constraint; identifiability is ensured by anchoring the initial level together with the likelihood, following standard practice in intrinsic Gaussian Markov random fields (IGMRFs).^20^ For regression coefficients we use independent Gaussian priors, $\beta_{j}∼N(0,\;1000)$ .

Posterior inference for $\theta_{\mathrm{obs}}=(\beta ,\{h_{0k}\})$ is conducted efficiently by integrated nested Laplace approximation (INLA),^11^ yielding draws or summaries of $H_{\mathrm{obs}}(t)$ and $h_{\mathrm{obs}}(t)$ on a pre-specified grid $\left\{t_{g}{\}}_{g=1}^{G}\subset [0,T_{⋆}\right]$ . The PEM can approximate any continuous hazard to arbitrary accuracy as the partition is refined, while the RW1 prior mitigates overfitting. In implementation, we specify the RW1 piecewise-exponential baseline on a grid that extends up to $T_{⋆}$ , while the likelihood uses only data observed on $\left[0,t_{\mathrm{obs}}\right]$ . Consequently, beyond the observation window the posterior of the baseline hazard is driven by the RW1 prior together with the boundary information inherited from the fitted portion of the curve at $t_{\mathrm{obs}}$ . This yields a smooth continuation of the observation-side component, represented by $\left\{H_{\mathrm{obs}}(t),h_{\mathrm{obs}}(t)\right\}$ for $t>t_{\mathrm{obs}}$ . This continuation is used to define the contrast with the external component and to enter the blended hazard in (3). It is not based on observed outcomes beyond $t_{\mathrm{obs}}$ ; rather, it is the model-based continuation of the observation-side component implied by the fitted piecewise exponential model and RW1 prior.

#### 2.4.2. External tail on $\left[t_{\mathrm{obs}},T_{⋆}\right]$ : Gompertz with anchor identifiability

As described in Section 2.1, the external component is used to represent longer-term survival using external evidence or externally elicited information. We adopt a Gompertz tail, which captures exponentially bending hazards common in mortality contexts

(7) $$h_{\mathrm{ext}}(t)=\rho e^{\gamma t},\;H_{\mathrm{ext}}(t)=\frac{\rho }{\gamma }(e^{\gamma t}-1),\;S_{\mathrm{ext}}(t)=\exp\left\{-\frac{\rho }{\gamma }(e^{\gamma t}-1)\right\}.$$

Here t denotes time since baseline (years). The Gompertz tail has two parameters: ρ>0 is the initial hazard level with $h_{\mathrm{ext}}(0)=\rho$ (units: year^−1^), and γ controls the exponential bending of the hazard function via $\partial \{\log\;h_{\mathrm{ext}}(t)\}/\partial t=\gamma$ (units: year^−1^); when γ>0 , the hazard function increases over time, and the hazard-doubling time is $T_{\mathrm{double}}=\ln\;2/\gamma$ .

The anchor level $S_{a}\in (0,1)$ is an externally elicited survival probability at the anchor time $t_{a}\in [t_{\mathrm{obs}},T_{⋆}]$ . In our framework, this anchor is not treated as observed outcome data beyond $t_{\mathrm{obs}}$ ; rather, it is treated as prior information used to identify the level of the external tail through the condition $S_{\mathrm{ext}}(t_{a})=S_{a}$ . More specifically, because no trial outcomes are observed beyond $t_{\mathrm{obs}}$ , the Gompertz parameters (ρ,γ) are not identifiable from the trial data alone.

We therefore enforce an anchor constraint at $t_{a}\in [t_{\mathrm{obs}},T_{⋆}]$ ,

(8) $$S_{\mathrm{ext}}(t_{a})=S_{a}\in (0,1),$$

treated as prior information rather than data. Condition (8) induces the deterministic mapping

(9) $$\rho (\gamma ,S_{a})=\gamma \;\frac{-\log\;S_{a}}{e^{\gamma t_{a}}-1},$$

so that identifiability is achieved by parameterizing through $\left(\gamma ,S_{a}\right)$ and defining ρ via (9) (see Appendix B for identifiability, positivity of $\rho (\gamma ,S_{a})$ , and the exponential limit as γ→0 ).

We place a weakly informative prior on the tail shape on the log scale,

$$\log\;\gamma ∼N(\mu_{\gamma },\sigma_{\gamma }^{2}),\;\mu_{\gamma }=\log\;(0.08),\sigma_{\gamma }^{2}=0.5^{2},$$

and encode anchor uncertainty with a Beta prior centered at the specified anchor level. Let $S_{a}^{\text{mean}}\in (0,1)$ denote the pre-specified anchor survival probability at time $t_{a}$ . We parameterize

$$S_{a}∼\mathrm{Beta}(\alpha ,\beta ),\;\alpha =\mathrm{ESS}\cdot S_{a}^{\text{mean}},\;\beta =\mathrm{ESS}\cdot (1-S_{a}^{\text{mean}}),$$

where ESS is the prior effective sample size: it controls how concentrated the prior is and can be interpreted as the number of pseudo-observations consistent with a Bernoulli model having success proportion $S_{a}^{\text{mean}}$ . Unless otherwise stated we set ESS=50 , which implies $E[S_{a}]=S_{a}^{\text{mean}}$ and $\mathrm{Var}(S_{a})=S_{a}^{\text{mean}}(1-S_{a}^{\text{mean}})/(\mathrm{ESS}+1)$ ; the maximal prior standard deviation is therefore $\sqrt{0.25/(50+1)}\approx 0.07$ at $S_{a}^{\text{mean}}=0.5$ .

We draw M=2000 independent samples $\{(\gamma^{\left(m\right)},S_{a}^{\left(m\right)}){\}}_{m=1}^{M}$ , map $\rho^{\left(m\right)}=\rho (\gamma^{\left(m\right)},S_{a}^{\left(m\right)})$ via (9), and propagate this uncertainty to $H_{\mathrm{ext}}(t)$ and $h_{\mathrm{ext}}(t)$ (Algorithm 1, Step 3).

This construction transparently separates the trial likelihood, which describes short-term behavior using observed data up to $t_{\mathrm{obs}}$ , from the prior information used to encode longer-term survival behavior through the anchor. In this sense, the anchor influences the external tail through prior specification rather than by contributing observed outcome data beyond the trial follow-up period. In all reported results, we enforce γ>0 (i.e. increasing Gompertz hazard), hence the prior distribution is defined on logγ ; general properties that hold for arbitrary γ∈R are deferred to Appendix B.

#### 2.4.3. Adaptive weighting function: monotone P-splines with temperature scaling

We estimate the time-varying weight π(t) by regressing the log-cumulative-hazard contrast

(10) $$z(t)=\log\;H_{\mathrm{obs}}(t)-\log\;H_{\mathrm{ext}}(t)$$

onto time t using a shape-constrained additive model (SCAM).^13^ Specifically, we fit a monotone-increasing P-spline

(11) $$\eta (t)=\sum_{j=1}^{J}\xi_{j}B_{j}(t),\;\xi_{j}\geq 0,$$

where $\left\{B_{j}\right\}$ are the monotonically positively increasing (mpi) P-spline basis functions,^13,21^ which ensure $B_{j}^{\prime }(t)\geq 0$ and the smoothing parameter is selected via restricted maximum likelihood (REML). Mapping η(t) through the logistic link yields the preliminary weight

(12) $$\pi_{\mathrm{raw}}(t)=\{1+\exp\;[-\eta (t)]{\}}^{-1}\in (0,1),$$

which is nondecreasing because $\eta^{\prime }(t)\geq 0$ and the logistic map is increasing.

To ensure a valid (nonnegative) blended hazard in (3), we temper the slope of π(t) by a scalar temperature parameter τ≥1 ,

(13) $$\pi_{\tau }(t)=\{1+\exp\;[-\eta (t)/\tau ]{\}}^{-1},\;\pi_{\tau }^{\prime }(t)=\frac{1}{\tau }\;\sigma^{\prime }\{\eta (t)/\tau \}\;\eta^{\prime }(t),$$

where $\sigma (u)=\{1+\exp\;(-u){\}}^{-1}$ and $\sigma^{\prime }(u)=\sigma (u)\{1-\sigma (u)\}$ . As τ→∞ , the drift term in (3) vanishes and $h_{\mathrm{blend}}(t)$ approaches the convex combination $(1-\pi_{\tau })h_{\mathrm{obs}}(t)+\pi_{\tau }h_{\mathrm{ext}}(t)\geq 0$ . We control the slope of $\pi^{\prime }(t)$ through the temperature parameter τ and select the smallest τ that satisfies the sufficient condition in Lemma A.5 (Appendix A), which guarantees ${\hat{h}}_{\mathrm{blend}}(t_{g})\geq 0$ for all grid points $\{t_{g}{\}}_{g=1}^{G}$ (see Corollary A.6).

For numerical stability and to avoid boundary artifacts at $t_{\mathrm{obs}}$ , we fit the monotone P-spline on the entire grid $t\in [0,T_{⋆}]$ . We do not hard-constrain the weight to be zero before $t_{\mathrm{obs}}$ ; hence π(t) may be slightly positive on $t\leq t_{\mathrm{obs}}$ . All evaluation metrics in Section 3 are reported on $\left[t_{\mathrm{obs}},T_{⋆}\right]$ .

Let $\hat{\eta }(t)$ denote the SCAM estimate of η(t) , and let $\pi_{\tau }(t)=\{1+\exp\;[-\hat{\eta }(t)/\tau ]{\}}^{-1}$ with the smallest τ≥1 such that $h_{\mathrm{blend}}(t_{g};\pi_{\tau })\geq 0$ on a grid $\left\{t_{g}{\}}_{g=1}^{G}\subset [0,T_{⋆}\right]$ . Denote by ${\hat{H}}_{\mathrm{obs}}(t)$ , ${\hat{h}}_{\mathrm{obs}}(t)$ and ${\hat{H}}_{\mathrm{ext}}(t)$ , ${\hat{h}}_{\mathrm{ext}}(t)$ the estimated cumulative hazards and hazards from the observation-side and the external tail components, respectively. The blended cumulative hazard and survival are then

(14) $${\hat{H}}_{\mathrm{blend}}(t)=(1-\pi_{\tau }(t))\;{\hat{H}}_{\mathrm{obs}}(t)+\pi_{\tau }(t)\;{\hat{H}}_{\mathrm{ext}}(t),\;{\hat{S}}_{\mathrm{blend}}(t)=\exp\;\{-{\hat{H}}_{\mathrm{blend}}(t)\},$$

and the blended hazard is

(15) $${\hat{h}}_{\mathrm{blend}}(t)=(1-\pi_{\tau }(t))\;{\hat{h}}_{\mathrm{obs}}(t)+\pi_{\tau }(t)\;{\hat{h}}_{\mathrm{ext}}(t)+\{{\hat{H}}_{\mathrm{ext}}(t)-{\hat{H}}_{\mathrm{obs}}(t)\}\;\pi_{\tau }^{\prime }(t),$$

where $\pi_{\tau }^{\prime }(t)=(1/\tau )\;\sigma^{\prime }\{\hat{\eta }(t)/\tau \}\;{\hat{\eta }}^{\prime }(t)$ . In implementation, $\pi_{\tau }^{\prime }(t)$ can be evaluated analytically from the SCAM fit or numerically by finite differences on $\left\{t_{g}\right\}$ . For interpretability, the weight can be read off the cumulative-hazard identity

(16) $$\pi_{\tau }(t)=\frac{{\hat{H}}_{\mathrm{blend}}(t)-{\hat{H}}_{\mathrm{obs}}(t)}{{\hat{H}}_{\mathrm{ext}}(t)-{\hat{H}}_{\mathrm{obs}}(t)}\;\text{whenever }{\hat{H}}_{\mathrm{ext}}(t)\neq {\hat{H}}_{\mathrm{obs}}(t).$$

**Table:**
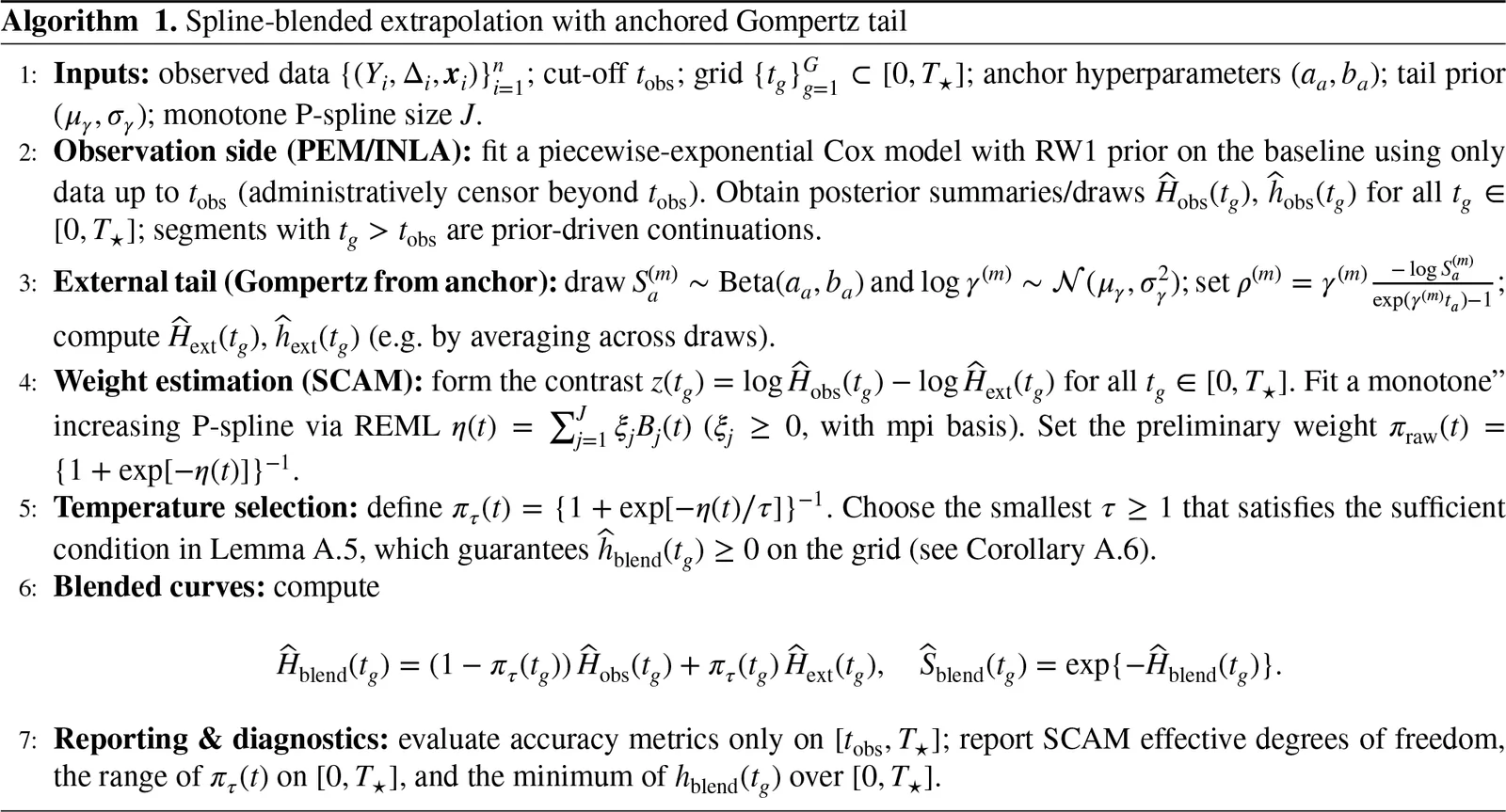


## 3. Simulation study

### 3.1. Design and evaluation metrics

We conducted a Monte Carlo study to assess the finite-sample performance of the proposed spline-blended extrapolation across different pre-specified experiments. Unless otherwise stated, models are trained on data up to $t_{\mathrm{obs}}=10$ years and evaluated through $T_{⋆}=20$ years. We simulate independent and identical distributed (i.i.d.) survival times for n=2000 subjects from four distributions with distinct tail behaviors (Exponential, Weibull, Log-normal, Log-logistic), each calibrated so that the 20’ year survival is 0.20 . Administrative censoring is imposed at $t_{\mathrm{obs}}=10$ and, among subjects with events before $t_{\mathrm{obs}}$ , additional independent censoring is applied to achieve a target overall censoring proportion c . External information is incorporated through an anchored Gompertz tail identified by a survival level at $t_{a}=T_{⋆}=20$ years. In Scenarios 1 and 2, we fix the anchor mean at $S_{a}=0.10$ across datasets, while the data-generating distributions are calibrated to have true 20-year survival $S_{\mathrm{true}}(20)=0.20$ . This deliberate mismatch is intended to mimic a realistic setting in which externally informed long-term survival input is informative but imperfect, as is often the case in HTA and clinical extrapolation where the long-horizon truth is unknown. Uncertainty is propagated by drawing M=2000 samples of $\left(S_{a},\gamma \right)$ from their priors (with $\log\;\gamma ∼N(\log\;0.08,\;0.5^{2})$ ) and constructing $S_{\mathrm{ext}}(t)$ accordingly. The full scenario settings are summarized in Appendix Table A1. We compare: (i) spline-blended method: on the observation side we fit a piecewise-exponential (Cox-type) model with unit time bins and an RW1 prior on the log-baseline using INLA. The time grid runs to $T_{⋆}$ , but only outcomes $\leq t_{\mathrm{obs}}$ enter the likelihood; beyond $t_{\mathrm{obs}}$ the RW1 smoothness prior provides a principled forward continuation, yielding an extrapolated observation-side component. The external component is an anchored Gompertz tail calibrated at the target horizon.

We then construct a time-varying weight by contrasting the two components on the cumulative-hazard scale, smoothing this contrast with a monotone P-spline over $\left[0,T_{⋆}\right]$ , and transforming the smoothed contrast into π(t) via a logistic link; no endpoints are forced, so $\pi (t_{\mathrm{obs}})$ and $\pi (T_{⋆})$ are data-driven. Consequently, the spline-blended curve is not constrained to satisfy $S_{\mathrm{blend}}(T_{⋆})=S_{\mathrm{ext}}(T_{⋆})$ ; when the learned horizon weight remains below 1, the spline curve ends between the observation-side continuation and the external tail. (ii) a beta-blended method that uses the same two components but a fixed Beta (3,3) transition on $\left[t_{\mathrm{obs}},T_{⋆}\right]$ , so that $\pi_{\beta }(T_{⋆})=1$ and therefore $S_{\mathrm{blend}}(T_{⋆})=S_{\mathrm{ext}}(T_{⋆})$ by construction; and (iii) four single-family parametric models (Exponential, Weibull, Log-normal, Log-logistic) fitted by MLE on data truncated at $t_{\mathrm{obs}}$ , without external information. Accordingly, the blended methods and the single-family parametric comparators are not intended to represent an equal-information comparison. The blended methods use external long-term anchor information in addition to the observed data, whereas the single-family parametric models are fitted using only the censored data observed up to $t_{\mathrm{obs}}$ . This design reflects a pragmatic HTA setting in which trial follow-up is limited but some external long-term input is available. It is also relevant to applied settings because right-censoring due to administrative study end, loss to follow-up, or withdrawal is common in clinical follow-up and must be accommodated in extrapolation practice.

Software choices mirror the reported results: short-term fits use INLA;^11^ parametric fits use flexsurv;^7^ Kaplan–Meier estimates and RMST computations are obtained using survival.^10^ RMST over $\left[t_{\mathrm{obs}},t\right]$ is computed via the trapezoidal rule following.^15^ Evaluation focuses on the generating survival $S_{\mathrm{true}}(t)$ and a fitted curve $\hat{S}(t)$ . We report the absolute survival error $AE(Surv)(t)=E_{S}(t)=|S_{\mathrm{true}}(t)-\hat{S}(t)|$ at t∈{15,20} ; the RMST error $AE(RMST)(t)=E_{R}(t)=|\int_{t_{\mathrm{obs}}}^{t}S_{\mathrm{true}}(u)\;du-\int_{t_{\mathrm{obs}}}^{t}\hat{S}(u)\;du|$ at t∈{15,20} ; the mean absolute error $\mathrm{MAE}=(T_{⋆}-t_{\mathrm{obs}}{)}^{-1}\int_{t_{\mathrm{obs}}}^{T_{⋆}}|S_{\mathrm{true}}(u)-\hat{S}(u)|\;du$ ; and the stability gap $\mathrm{FD}=|E_{R}(20)-E_{R}(15)|$ . Each configuration is replicated R=1000 times, and we summarize Monte Carlo means and standard deviations (SDs).

The three experiments align exactly with the results tables and figures. In Scenario 1 (data from a Weibull distribution) the overall censoring level varies with c∈{10%,20%,30%,40%,50%} and the scenario compares spline-blended, beta-blended, and the four parametric models. In Scenario 2 (data not from a Weibull distribution), the censoring level is fixed c=50% and the scenario uses Exponential, Log-normal, and Log-logistic data-generating mechanisms, again comparing spline-blended, beta-blended, and the four parametric models. In Scenario 3 (sensitivity experiment) the data-generating mechanism is fixed to be Weibull and the overall censoring is fixed at 30% , the anchor mean is varied $S_{a}\in \{0.06,0.08,\;0.10,0.12,0.14,0.16\}$ , and the observation cutoff is varied $t_{\mathrm{obs}}\in \{8,10,12\}$ , the scenario evaluates only the spline-blended method with R=1000 replicates.

### 3.2. Results

#### 3.2.1. Scenario 1—Model comparison across censoring rates

We evaluate extrapolation from 0–10 years to 20 years under a scenario where the data-generating (true) survival curve is Weibull, calibrated to S(20)=0.20 ; note that this “Weibull scenario” refers to the truth, while the external tail used for blending remains Gompertz and is anchored at 20 years. Across R=1000 Monte Carlo datasets ( n=2000 ) with overall censoring at 10–50% (see Section 3.1), we compare our spline-blended method with the beta-blended method and four parametric models. Accuracy is assessed by AE(Surv) at 15/20 years, AE(RMST) from 10 to 15/20 years, MAE over 10–20 years, and the stability gap FD. Results are summarized in Table 2 and Figure 2.

**Figure 2.**
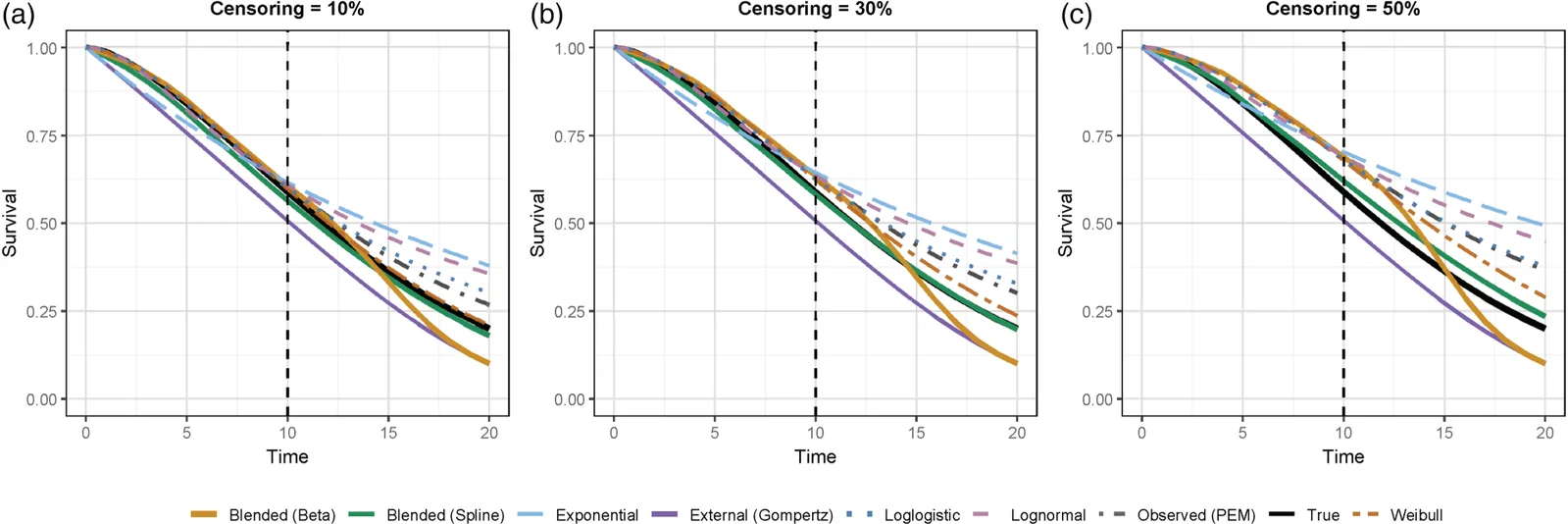
Survival extrapolation under censoring levels 10%, 30%, and 50% in Scenario 1.

**Table 2.** Performance by censoring level in Scenario 1 (Weibull).

		AE (Surv)		AE (RMST)		MAE	FD
Censoring	Model	@15y	@20y	@15y	@20y	( ± SD)	( ± SD)
0.1	Blended (Spline)	0.026	0.026	0.135	0.262	0.026 ± 0.010	0.127 ± 0.047
	Blended (Beta)	0.037	0.100	0.085	0.676	0.052 ± 0.003	0.591 ± 0.015
	Exponential	0.112	0.169	0.424	1.337	0.113 ± 0.012	0.913 ± 0.063
	Weibull	0.013	0.014	0.057	0.125	0.012 ± 0.009	0.069 ± 0.054
	Log-normal	0.088	0.146	0.324	1.118	0.091 ± 0.014	0.794 ± 0.079
	Log-logistic	0.050	0.095	0.177	0.698	0.054 ± 0.013	0.521 ± 0.071
0.2	Blended (Spline)	0.025	0.025	0.132	0.257	0.026 ± 0.010	0.125 ± 0.047
	Blended (Beta)	0.036	0.100	0.082	0.673	0.052 ± 0.003	0.591 ± 0.015
	Exponential	0.113	0.171	0.429	1.348	0.114 ± 0.011	0.919 ± 0.060
	Weibull	0.012	0.013	0.056	0.123	0.012 ± 0.009	0.068 ± 0.054
	Log-normal	0.088	0.147	0.329	1.126	0.092 ± 0.013	0.798 ± 0.078
	Log-logistic	0.051	0.095	0.181	0.705	0.055 ± 0.012	0.524 ± 0.070
0.3	Blended (Spline)	0.012	0.013	0.058	0.125	0.012 ± 0.008	0.068 ± 0.044
	Blended (Beta)	0.025	0.100	0.038	0.605	0.055 ± 0.003	0.567 ± 0.048
	Exponential	0.144	0.204	0.582	1.669	0.146 ± 0.011	1.088 ± 0.061
	Weibull	0.029	0.026	0.143	0.265	0.028 ± 0.014	0.125 ± 0.082
	Log-normal	0.116	0.173	0.461	1.391	0.119 ± 0.014	0.929 ± 0.082
	Log-logistic	0.075	0.116	0.306	0.933	0.079 ± 0.013	0.627 ± 0.075
0.4	Blended (Spline)	0.015	0.011	0.066	0.113	0.013 ± 0.009	0.050 ± 0.041
	Blended (Beta)	0.012	0.100	0.109	0.524	0.063 ± 0.004	0.415 ± 0.081
	Exponential	0.183	0.245	0.763	2.059	0.184 ± 0.011	1.296 ± 0.062
	Weibull	0.063	0.053	0.307	0.567	0.060 ± 0.017	0.260 ± 0.108
	Log-normal	0.149	0.206	0.622	1.716	0.151 ± 0.014	1.094 ± 0.088
	Log-logistic	0.106	0.144	0.459	1.218	0.109 ± 0.014	0.759 ± 0.084
0.5	Blended (Spline)	0.040	0.029	0.192	0.328	0.036 ± 0.013	0.137 ± 0.070
	Blended (Beta)	0.008	0.100	0.228	0.429	0.074 ± 0.005	0.201 ± 0.085
	Exponential	0.227	0.294	0.974	2.521	0.228 ± 0.011	1.547 ± 0.063
	Weibull	0.104	0.092	0.508	0.958	0.100 ± 0.018	0.450 ± 0.125
	Log-normal	0.188	0.246	0.812	2.109	0.190 ± 0.015	1.297 ± 0.096
	Log-logistic	0.144	0.178	0.643	1.570	0.145 ± 0.015	0.927 ± 0.094

Notes: Errors are computed on the extrapolation window $\left[t_{\mathrm{obs}},T_{⋆}\right]$ ; here $t_{\mathrm{obs}}=10$ y and $T_{⋆}=20$ y. The simulated event times follow a Weibull-based scenario calibrated to S(20)=0.20 . AE(Surv) = absolute survival error at 15y and 20y; AE(RMST) = absolute error in restricted mean survival time from $t_{\mathrm{obs}}$ up to 15y and 20y. MAE = mean absolute error of survival over $\left[t_{\mathrm{obs}},T_{⋆}\right]$ ; FD = $|{\mathrm{AE}(\mathrm{RMST})}_{20\text{y}}-{\mathrm{AE}(\mathrm{RMST})}_{15\text{y}}|$ . “ ± SD” reports Monte Carlo mean ± standard deviation across replicates. “Censoring” denotes the overall proportion censored at study end. Models: *Blended (Spline)* uses an adaptive monotone P-spline weight; *Blended (Beta)* uses a fixed-schedule Beta (3,3) weight on $\left[t_{\mathrm{obs}},T_{⋆}\right]$ ; *Exponential, Weibull, Log-normal and Log-logistic* are parametric models.

Table 2 reports Monte Carlo means (and SDs). The proposed spline-blended approach achieves the smallest or near-smallest MAE at every censoring level and maintains low FD as censoring increases. At 30% censoring it attains MAE=0.012(±0.008) and FD=0.068(±0.044) , compared with 0.055(±0.003) and 0.567(±0.048) for the beta-blended method and 0.028(±0.014) and 0.125(±0.082) for the parametric Weibull. Even at 50% censoring the spline-blended method remains competitive ( MAE=0.036 , FD=0.137 ), whereas the MAE of the beta-blended method and parametric Weibull method increases to 0.074 and 0.100 , respectively. Under lighter censoring (10%–20%), the fitted Weibull model remains the best-performing method, which is reassuring under correct parametric specification. The spline-blended method overtakes it mainly once censoring becomes moderate to high (30%–50%), when long-horizon extrapolation is less well determined by the observed trial window alone.

The beta-blended method shows a persistent survival error at 20 years of 0.100 across all censoring levels, reflecting its constraint $S_{\text{blend}}(20)=S_{\text{ext}}(20)$ . This behavior is expected given the simulation design: in Scenario 1, the external tail is anchored at 20-year survival $S_{a}=0.10$ , whereas the generating truth has $S_{\mathrm{true}}(20)=0.20$ . Hence the Beta comparator is mechanically pulled to the anchor at 20 years. By contrast, the spline-blended method is not endpoint constrained, and therefore may remain above the external tail when the learned horizon weight is below 1. In this setting, the fact that the spline curve ends higher than the external Gompertz tail is not a failure to respect the external input; rather, it reflects adaptive partial borrowing from an informative but imperfect anchor. Other misspecified parametric models (Exponential, Log-normal, Log-logistic) incur substantially larger long-horizon RMST errors and FD, especially as the censoring level increases. Figure 2 visualizes three censoring levels (10%, 30%, 50%): the spline-blended method transitions smoothly from the short-term fit to the anchored tail and tracks the scenario closely beyond $t_{\mathrm{obs}}$ , whereas the beta-blended method drifts toward the anchor at 20 y and the misspecified parametric shows large errors as the time-frame increases.

#### 3.2.2. Scenario 2 – Model comparison across different data-generating processes

The blended methods make use of external anchor information, whereas the single-family parametric comparators rely only on the observed data up to $t_{\mathrm{obs}}$ . The advantage of the spline-blended method therefore reflects not only flexibility of the adaptive transition, but also the fact that it can borrow from additional long-term information when the observed trial window alone is insufficient to determine the tail reliably. We next examine performance when the data-generating survival curve is not Weibull, considering Exponential, Log-normal, and Log-logistic models while keeping the overall setup as in Section 3.1. (In the table below, “Scenario” denotes the data-generating distribution, and “Model” the model used to fit the data on the time frame of [0-10] years.) We compare the proposed spline-blended method, the beta-blended method, and four parametric models (Exponential, Weibull, Log-normal, Log-logistic) that use no external information.

Table 3 reports Monte Carlo means (MAE/FD as mean ± SD) under the three non-Weibull data-generating processes. The adaptive P-spline attains the lowest overall error in every scenario, with MAE =0.050(±0.006) for data from the Exponential model, 0.014(±0.008) for Log-normal, and 0.035(±0.007) for Log-logistic. Its long-horizon survival errors at 20 years remain small: 0.006 , 0.016 , and 0.019 , respectively. The beta-blended method again exhibits the characteristic that the survival error at 20 years is 0.10 (by construction) across all three scenarios. This again reflects the fixed anchor mean $S_{a}=0.10$ together with the Beta method’s endpoint constraint $S_{\mathrm{blend}}(20)=S_{\mathrm{ext}}(20)$ , whereas the spline method is free to end above the external tail when the learned horizon weight remains below 1. Accordingly, the smaller 20-year errors of the spline method reflect adaptive partial borrowing rather than exact matching to the external anchor. It delivers larger RMST errors at one or both horizons in Log-normal ( 0.546 at 20 years vs. 0.130 for the spline-blended method) and Log-logistic ( 0.361 at 20 years vs. 0.113 for the spline-blended method), while performing similarly in the Exponential case (RMST at 20 years of 0.278 vs. 0.279 for the spline-blended method). In terms of stability, the spline weight yields small FD (Exponential 0.077 , Log-normal 0.077 ), and although the beta-blended method has a slightly smaller FD in the case of data from the Log-logistic model ( 0.095 vs. 0.128 ), this comes with markedly worse AE/RMST. Misspecified single-family parametric fits are uniformly less accurate (e.g. under simulations from the Exponential model, MAE =0.116 for Weibull and 0.126 for Exponential vs. 0.050 for spline; under Log-normal, the best single-family alternative, Log-logistic, still has MAE =0.041 ). Overall, allowing the blending weight to adapt over time provides robust extrapolation across heterogeneous tail shapes without relying on a correct global parametric specification.

**Table 3.** Performance by data-generating process in scenario 2 (non-weibull).

		AE (Surv)		AE (RMST)		MAE	FD
Scenario	Model	@15y	@20y	@15y	@20y	(mean ± SD)	(mean ± SD)
Exponential	Blended (Spline)	0.057	0.006	0.355	0.279	0.050 ± 0.006	0.077 ± 0.035
	Blended (Beta)	0.045	0.100	0.410	0.278	0.081 ± 0.004	0.134 ± 0.064
	Exponential	0.128	0.122	0.632	1.235	0.126 ± 0.011	0.604 ± 0.057
	Weibull	0.118	0.108	0.593	1.123	0.116 ± 0.014	0.530 ± 0.086
	Log-normal	0.188	0.218	0.865	1.989	0.188 ± 0.012	1.123 ± 0.073
	Log-logistic	0.153	0.171	0.723	1.598	0.153 ± 0.012	0.875 ± 0.070
Log-normal	Blended (Spline)	0.015	0.016	0.058	0.130	0.014 ± 0.008	0.077 ± 0.047
	Blended (Beta)	0.026	0.100	0.069	0.546	0.061 ± 0.003	0.478 ± 0.091
	Exponential	0.275	0.345	1.156	2.964	0.271 ± 0.011	1.808 ± 0.065
	Weibull	0.035	0.118	0.082	0.685	0.065 ± 0.010	0.603 ± 0.204
	Log-normal	0.091	0.078	0.441	0.823	0.086 ± 0.018	0.381 ± 0.107
	Log-logistic	0.041	0.020	0.257	0.317	0.041 ± 0.016	0.084 ± 0.062
Log-logistic	Blended (Spline)	0.035	0.019	0.237	0.113	0.035 ± 0.007	0.128 ± 0.045
	Blended (Beta)	0.022	0.100	0.294	0.361	0.070 ± 0.005	0.095 ± 0.071
	Exponential	0.199	0.204	0.915	1.947	0.192 ± 0.011	1.032 ± 0.061
	Weibull	0.038	0.030	0.287	0.148	0.044 ± 0.008	0.159 ± 0.072
	Log-normal	0.132	0.125	0.633	1.255	0.128 ± 0.015	0.622 ± 0.090
	Log-logistic	0.091	0.073	0.473	0.818	0.088 ± 0.015	0.345 ± 0.079

#### 3.2.3. Scenario 3—Sensitivity to observation window and anchor level

We assess how extrapolation accuracy varies with the observation cutoff $t_{\mathrm{obs}}\in \{8,10,12\}$ years and the anchor mean $S_{a}\in \{0.10,0.12,0.14,0.16,0.18,0.20\}$ under a Weibull data-generating curve calibrated to S(20)=0.20 . Each cell uses R=1000 Monte Carlo replicates. Table 4 reports the main comparison against the adaptive spline-blended method, the fixed-schedule Beta method, and the correctly specified Weibull model; the full results including the other parametric comparators are reported in the Supplementary Tables (Supplementary Table S1A – S1C).

**Table 4.** Effect of observation cutoff $t_{\mathrm{obs}}$ and anchor mean $S_{a}$ in scenario 3 (sensitivity), with comparison to the beta and weibull models.

			AE (Surv)		AE (RMST)		MAE	FD
$t_{\mathrm{obs}}$ (y)	$S_{a}$	Model	@15y	@20y	@15y	@20y	(mean ± SD)	(mean ± SD)
8	0.10	Blended (Spline)	0.013 ± 0.009	0.011 ± 0.008	0.082 ± 0.060	0.130 ± 0.095	0.012 ± 0.007	0.050 ± 0.038
		Blended (Beta)	0.043 ± 0.007	0.100 ± 0.001	0.095 ± 0.061	0.731 ± 0.052	0.053 ± 0.003	0.637 ± 0.022
		Weibull	0.030 ± 0.019	0.028 ± 0.020	0.198 ± 0.114	0.330 ± 0.216	0.029 ± 0.017	0.137 ± 0.105
	0.12	Blended (Spline)	0.015 ± 0.011	0.014 ± 0.010	0.081 ± 0.060	0.145 ± 0.108	0.013 ± 0.008	0.066 ± 0.049
		Blended (Beta)	0.021 ± 0.007	0.080 ± 0.001	0.057 ± 0.042	0.531 ± 0.053	0.042 ± 0.005	0.475 ± 0.073
		Weibull	0.030 ± 0.019	0.028 ± 0.020	0.198 ± 0.114	0.330 ± 0.216	0.029 ± 0.017	0.137 ± 0.105
	0.14	Blended (Spline)	0.024 ± 0.014	0.021 ± 0.012	0.127 ± 0.080	0.231 ± 0.140	0.019 ± 0.011	0.104 ± 0.062
		Blended (Beta)	0.007 ± 0.005	0.060 ± 0.001	0.105 ± 0.065	0.334 ± 0.058	0.033 ± 0.006	0.232 ± 0.115
		Weibull	0.030 ± 0.019	0.028 ± 0.020	0.198 ± 0.114	0.330 ± 0.216	0.029 ± 0.017	0.137 ± 0.105
	0.16	Blended (Spline)	0.034 ± 0.014	0.031 ± 0.013	0.192 ± 0.085	0.344 ± 0.148	0.029 ± 0.012	0.152 ± 0.064
		Blended (Beta)	0.017 ± 0.008	0.040 ± 0.001	0.179 ± 0.074	0.145 ± 0.056	0.028 ± 0.008	0.107 ± 0.080
		Weibull	0.030 ± 0.019	0.028 ± 0.020	0.198 ± 0.114	0.330 ± 0.216	0.029 ± 0.017	0.137 ± 0.105
	0.18	Blended (Spline)	0.045 ± 0.014	0.041 ± 0.013	0.263 ± 0.084	0.466 ± 0.146	0.039 ± 0.012	0.203 ± 0.063
		Blended (Beta)	0.034 ± 0.009	0.020 ± 0.002	0.253 ± 0.076	0.057 ± 0.043	0.028 ± 0.008	0.197 ± 0.049
		Weibull	0.030 ± 0.019	0.028 ± 0.020	0.198 ± 0.114	0.330 ± 0.216	0.029 ± 0.017	0.137 ± 0.105
	0.20	Blended (Spline)	0.056 ± 0.014	0.051 ± 0.013	0.333 ± 0.080	0.586 ± 0.141	0.049 ± 0.012	0.253 ± 0.062
		Blended (Beta)	0.050 ± 0.009	0.001 ± 0.001	0.322 ± 0.077	0.224 ± 0.056	0.035 ± 0.009	0.098 ± 0.024
		Weibull	0.030 ± 0.019	0.028 ± 0.020	0.198 ± 0.114	0.330 ± 0.216	0.029 ± 0.017	0.137 ± 0.105
10	0.10	Blended (Spline)	0.012 ± 0.009	0.013 ± 0.009	0.058 ± 0.042	0.125 ± 0.086	0.012 ± 0.008	0.068 ± 0.044
		Blended (Beta)	0.025 ± 0.008	0.100 ± 0.001	0.038 ± 0.029	0.605 ± 0.037	0.055 ± 0.003	0.567 ± 0.048
		Weibull	0.029 ± 0.016	0.026 ± 0.017	0.143 ± 0.069	0.265 ± 0.161	0.028 ± 0.014	0.125 ± 0.082
	0.12	Blended (Spline)	0.010 ± 0.007	0.009 ± 0.007	0.040 ± 0.030	0.086 ± 0.062	0.009 ± 0.006	0.047 ± 0.035
		Blended (Beta)	0.010 ± 0.007	0.080 ± 0.001	0.058 ± 0.040	0.446 ± 0.036	0.043 ± 0.003	0.388 ± 0.074
		Weibull	0.026 ± 0.016	0.022 ± 0.017	0.129 ± 0.069	0.233 ± 0.161	0.025 ± 0.015	0.109 ± 0.088
	0.14	Blended (Spline)	0.014 ± 0.010	0.010 ± 0.008	0.062 ± 0.042	0.108 ± 0.082	0.012 ± 0.009	0.048 ± 0.040
		Blended (Beta)	0.009 ± 0.007	0.060 ± 0.001	0.099 ± 0.050	0.285 ± 0.036	0.034 ± 0.005	0.186 ± 0.085
		Weibull	0.026 ± 0.016	0.022 ± 0.017	0.129 ± 0.069	0.233 ± 0.161	0.025 ± 0.015	0.109 ± 0.088
	0.16	Blended (Spline)	0.025 ± 0.011	0.018 ± 0.011	0.113 ± 0.047	0.197 ± 0.102	0.022 ± 0.010	0.084 ± 0.055
		Blended (Beta)	0.022 ± 0.010	0.040 ± 0.001	0.143 ± 0.052	0.126 ± 0.041	0.027 ± 0.006	0.074 ± 0.058
		Weibull	0.026 ± 0.016	0.022 ± 0.017	0.129 ± 0.069	0.233 ± 0.161	0.025 ± 0.015	0.109 ± 0.088
	0.18	Blended (Spline)	0.036 ± 0.011	0.028 ± 0.011	0.166 ± 0.046	0.300 ± 0.100	0.033 ± 0.010	0.134 ± 0.055
		Blended (Beta)	0.034 ± 0.010	0.020 ± 0.001	0.182 ± 0.053	0.040 ± 0.028	0.026 ± 0.007	0.142 ± 0.038
		Weibull	0.026 ± 0.016	0.022 ± 0.017	0.129 ± 0.069	0.233 ± 0.161	0.025 ± 0.015	0.109 ± 0.088
	0.20	Blended (Spline)	0.047 ± 0.011	0.038 ± 0.011	0.219 ± 0.044	0.403 ± 0.098	0.043 ± 0.010	0.185 ± 0.055
		Blended (Beta)	0.046 ± 0.010	0.001 ± 0.001	0.218 ± 0.054	0.178 ± 0.041	0.032 ± 0.007	0.040 ± 0.017
		Weibull	0.026 ± 0.016	0.022 ± 0.017	0.129 ± 0.069	0.233 ± 0.161	0.025 ± 0.015	0.109 ± 0.088
12	0.10	Blended (Spline)	0.015 ± 0.009	0.021 ± 0.010	0.042 ± 0.025	0.145 ± 0.078	0.017 ± 0.010	0.103 ± 0.053
		Blended (Beta)	0.009 ± 0.007	0.100 ± 0.001	0.063 ± 0.031	0.483 ± 0.030	0.057 ± 0.002	0.421 ± 0.060
		Weibull	0.028 ± 0.014	0.023 ± 0.015	0.083 ± 0.039	0.199 ± 0.116	0.026 ± 0.014	0.117 ± 0.076
	0.12	Blended (Spline)	0.008 ± 0.006	0.012 ± 0.009	0.021 ± 0.016	0.076 ± 0.058	0.009 ± 0.006	0.056 ± 0.046
		Blended (Beta)	0.016 ± 0.009	0.080 ± 0.001	0.080 ± 0.034	0.352 ± 0.032	0.046 ± 0.003	0.273 ± 0.065
		Weibull	0.028 ± 0.014	0.023 ± 0.015	0.083 ± 0.039	0.199 ± 0.116	0.026 ± 0.014	0.117 ± 0.076
	0.14	Blended (Spline)	0.012 ± 0.007	0.008 ± 0.007	0.035 ± 0.021	0.069 ± 0.049	0.011 ± 0.006	0.039 ± 0.034
		Blended (Beta)	0.024 ± 0.011	0.060 ± 0.001	0.095 ± 0.035	0.224 ± 0.032	0.036 ± 0.004	0.129 ± 0.066
		Weibull	0.028 ± 0.014	0.023 ± 0.015	0.083 ± 0.039	0.199 ± 0.116	0.026 ± 0.014	0.117 ± 0.076
	0.16	Blended (Spline)	0.022 ± 0.009	0.012 ± 0.008	0.066 ± 0.024	0.124 ± 0.069	0.019 ± 0.008	0.062 ± 0.042
		Blended (Beta)	0.032 ± 0.012	0.040 ± 0.001	0.109 ± 0.036	0.101 ± 0.033	0.028 ± 0.005	0.055 ± 0.041
		Weibull	0.028 ± 0.014	0.023 ± 0.015	0.083 ± 0.039	0.199 ± 0.116	0.026 ± 0.014	0.117 ± 0.076
	0.18	Blended (Spline)	0.033 ± 0.009	0.021 ± 0.010	0.098 ± 0.024	0.204 ± 0.076	0.029 ± 0.009	0.106 ± 0.051
		Blended (Beta)	0.039 ± 0.012	0.020 ± 0.001	0.121 ± 0.037	0.033 ± 0.023	0.026 ± 0.007	0.090 ± 0.027
		Weibull	0.028 ± 0.014	0.023 ± 0.015	0.083 ± 0.039	0.199 ± 0.116	0.026 ± 0.014	0.117 ± 0.076
	0.20	Blended (Spline)	0.044 ± 0.009	0.031 ± 0.010	0.131 ± 0.023	0.287 ± 0.075	0.040 ± 0.009	0.157 ± 0.053
		Blended (Beta)	0.046 ± 0.013	0.001 ± 0.001	0.133 ± 0.037	0.140 ± 0.034	0.030 ± 0.007	0.010 ± 0.007
		Weibull	0.028 ± 0.014	0.023 ± 0.015	0.083 ± 0.039	0.199 ± 0.116	0.026 ± 0.014	0.117 ± 0.076

First, for the adaptive spline-blended method, performance is best when the anchor lies in a moderate range close to, but not necessarily exactly equal to, the long-horizon truth. With $t_{\mathrm{obs}}=10$ years, the mean MAE decreases from 0.012 at $S_{a}=0.10$ to 0.009 at $S_{a}=0.12$ , remains low at 0.012 for $S_{a}=0.14$ , and then increases to 0.022 , 0.033 , and 0.043 as $S_{a}$ moves to 0.16 , 0.18 , and 0.20 , respectively. The same pattern appears in AE(Surv)(20), which is 0.013 at $S_{a}=0.10$ , 0.009 at $S_{a}=0.12$ , 0.010 at $S_{a}=0.14$ , and then rises to 0.018 , 0.028 , and 0.038 . With $t_{\mathrm{obs}}=12$ , the best operating region is again around $S_{a}=0.12$ – 0.14 : MAE is 0.009 at $S_{a}=0.12$ and 0.011 at $S_{a}=0.14$ , while AE(Surv)(20) reaches its minimum of 0.008 at $S_{a}=0.14$ . With the shorter observation window $t_{\mathrm{obs}}=8$ , the spline method performs best at the lower end of the grid, with MAE =0.012 and AE(Surv)(20) =0.011 at $S_{a}=0.10$ . Second, the spline method improves on the correctly specified Weibull benchmark over a fairly broad range of anchor choices, rather than only at one finely tuned value. For example, when $t_{\mathrm{obs}}=10$ , the Weibull model has MAE =0.028 , whereas the spline method attains MAE values of 0.012 , 0.009 , 0.012 , and 0.022 for $S_{a}=0.10,0.12,0.14,$ and 0.16 , respectively. When $t_{\mathrm{obs}}=12$ , the Weibull MAE is 0.026 , while the spline method yields 0.017 , 0.009 , 0.011 , and 0.019 over the same anchor range. Thus, the adaptive spline approach remains competitive over a nontrivial neighborhood of plausible external inputs, rather than requiring exact anchor specification to improve on direct parametric extrapolation.

Third, the fixed-schedule Beta method behaves differently because its endpoint is constrained to satisfy $S_{\mathrm{blend}}(20)=S_{\mathrm{ext}}(20)$ . As $S_{a}$ approaches the true 20-year survival level of 0.20 , its AE(Surv)(20) decreases mechanically from about 0.10 at $S_{a}=0.10$ to essentially zero at $S_{a}=0.20$ . In that exact-match setting, the Beta method can indeed be very strong, and this is visible in Table 4. However, this advantage rests on a highly idealized assumption: namely, that the analyst knows in advance that the externally supplied long-term survival level is exactly equal to the true long-term survival of the target population. In real HTA and clinical-trial extrapolation, the difficulty is precisely that the long-horizon truth is unknown, while external information is informative but cannot be pre-verified to equal the truth exactly. For this reason, the strongest performance of the Beta method should be interpreted as arising under an oracle-like matching condition. Our motivation for adaptive partial borrowing is to avoid requiring that exact alignment in advance, while still allowing the external input to influence long-term behavior. In absolute terms, across the 18 spline settings considered here, AE(Surv)(20) ranges from 0.008 (at $t_{\mathrm{obs}}=12$ , $S_{a}=0.14$ ) to 0.051 (at $t_{\mathrm{obs}}=8$ , $S_{a}=0.20$ ); MAE ranges from 0.009 (at $t_{\mathrm{obs}}=10$ , $S_{a}=0.12$ , and at $t_{\mathrm{obs}}=12$ , $S_{a}=0.12$ ) to 0.049 (at $t_{\mathrm{obs}}=8$ , $S_{a}=0.20$ ); AE(RMST)(20) ranges from 0.069 (at $t_{\mathrm{obs}}=12$ , $S_{a}=0.14$ ) to 0.586 (at $t_{\mathrm{obs}}=8$ , $S_{a}=0.20$ ); and FD ranges from 0.039 (at $t_{\mathrm{obs}}=12$ , $S_{a}=0.14$ ) to 0.253 (at $t_{\mathrm{obs}}=8$ , $S_{a}=0.20$ ). Overall, the best operating region for the adaptive spline method is around $S_{a}=0.12$ – 0.14 , with the precise optimum depending slightly on the amount of observed follow-up and on the metric of interest.

As an additional empirical reference, we also compared our approach with survextrap^22^ in the Weibull-based simulation setting under 30% and 50% censoring (Supplementary Table S1D). The results show that the adaptive spline-blended method remained more accurate than survextrap in this setting, especially in the long-horizon metrics that are most central to our study. Under 30% censoring, the spline-blended method had smaller AE(Surv) at 20 years, smaller AE(RMST) at 20 years, and lower MAE than survextrap; the same general pattern was also seen under 50% censoring. At the same time, survextrap remained substantially more competitive than the simpler fixed parametric baselines, indicating that methods incorporating external information can indeed offer meaningful gains when follow-up is limited. In particular, survextrap is designed as a broader joint Bayesian synthesis framework, whereas our method is more targeted toward adaptive cumulative-hazard blending with explicit control of the transition between the observation-side and external components. The supplementary results therefore help illustrate that, in the present simulation setting, the proposed spline-blended approach can provide a more accurate and robust extrapolation profile, while survextrap remains an important and relevant comparator within the wider class of externally informed survival-extrapolation methods.

## 4. Real data application

### 4.1. Data description

To further investigate the empirical performance of our method on larger-scale datasets with longer follow-up, we analyze data from the Surveillance, Epidemiology, and End Results (SEER) Program. SEER is a publicly available cancer registry of the National Cancer Institute that collects and publishes cancer incidence and survival data from population-based cancer registries.^17^ The data is available for research purposes upon request through the SEER website (https://seer.cancer.gov/). We analyze the SEER9 database, which covers cancer registries from nine geographic areas in the United States from 1973 to 2021. Our analysis focuses on three cancer types: colorectal, melanoma, and pancreatic cancer. The study population includes adult patients (aged at least 20 years at diagnosis) with advanced or metastatic disease as their first primary malignancy. Advanced disease is defined using the SEER historic stage A classification of “distant” stage, which indicates cancer that has spread from the primary site to distant organs or tissues. We excluded patients who had incomplete event indicators (unknown death) or were diagnosed with cancer at autopsy. Due to the significant differences in survival outcomes by age at diagnosis across all three cancer types, with higher ages consistently associated with lower survival rates, we stratify each cancer cohort into three age groups (20 to 59 years, 60 to 69 years, and 70 years and older), resulting in nine distinct analysis cohorts.

For evaluation, we mimic limited follow-up by administratively censoring survivors at 3 years and extrapolating to 10 years. We use the Kaplan–Meier estimates as a practical long-horizon benchmark under censoring, while recognizing that the long-term truth is not directly known at the time extrapolation decisions are made.^23^ External information is specified using historical survival rates from previous SEER statistics,^17^ clinical prediction tools,^24^ and previous survival extrapolation studies,^23,25^ with target values chosen to allow realistic departures from the later observed 10-year Kaplan–Meier estimate-based benchmark.

In particular, our illustration is intended to reflect a common HTA setting in which long-term external targets may be somewhat optimistic relative to later real-world survival. Such optimism can arise because long-term expectations are often informed by efficacy results from selected trial populations, specialist-center experience, or favorable assumptions about long-term tail behavior, whereas later registry-based outcomes reflect broader patient populations, variable treatment adherence, and evolving subsequent-care pathways. Accordingly, in most cohorts the external 10-year targets are set above the later observed 10-year survival in SEER, while still remaining clinically plausible. For the all-ages cohorts these targets are 9% (colorectal), 20% (melanoma), and 2% (pancreatic), with age-specific values in Table 5.

**Table 5.** Characteristics and outcomes of SEER cancer registry cohorts.

				Observed 10-y outcomes		
Cancer	Age group (y)	n	Censoring Prop. (%)	RMST (months)	Survival (%)	External info. (%)
Colorectal	20–59	14{,}731	8.97	11.89	9.91	15.00
	60–69	12{,}604	4.36	7.23	6.03	9.00
	70+	12{,}935	2.04	4.33	3.61	5.00
	All	40{,}270	5.30	7.97	6.64	9.00
Melanoma	20–59	1{,}506	19.99	26.28	21.90	25.00
	60–69	852	15.61	19.55	16.29	20.00
	70+	821	8.04	11.35	9.46	15.00
	All	3{,}179	15.73	20.69	17.24	20.00
Pancreas	20–59	6{,}732	2.76	3.41	2.84	3.50
	60–69	7{,}822	1.20	1.48	1.23	2.50
	70+	7{,}459	0.52	0.68	0.57	0.50
	All	22{,}013	1.45	1.80	1.50	2.00

Notes: Results are presented by cancer type and age group (in years). n = number of patients in each cohort; Censoring Prop. = percentage of patients with right-censored survival times; Observed 10-y RMST = restricted mean survival time (in months) calculated from Kaplan-Meier estimates over 10 years; Observed 10-y Survival = survival probability at 10 years from Kaplan-Meier estimates; External Info represents the survival probability at 10 years provided as external information. The “All” category represents the combined analysis of all age groups within each cancer type.

Table 5 summarizes the cohort characteristics and 10-year outcomes. Colorectal cancer has the largest sample ( n=40,270 ), followed by pancreatic cancer ( n=22,013 ) and melanoma ( n=3,179 ). Overall censoring is highest in melanoma (15.73%), moderate in colorectal cancer (5.30%), and minimal in pancreatic cancer (1.45%). Consistent with disease severity, the observed 10-year survival and RMST (months) for the all-ages cohorts are 17.24% and 20.69 for melanoma, 6.64% and 7.97 for colorectal cancer, and 1.50% and 1.80 for pancreatic cancer.

### 4.2. Results of SEER dataset

Figure 3 displays Kaplan–Meier estimates, the observation-side Cox model with PEM estimated using data up to 36 months (plotted with its RW1 prior-driven continuation beyond 36 months), the anchored Gompertz tail (‘External’), and the proposed spline-blended curve; the vertical dashed line marks the 3-year administrative cutoff. The external Gompertz curve is shown on the full time axis for transparency, but it should not be interpreted as a stand-alone fit to the observed window. Rather, it is a long-horizon reference component identified by the external anchor, and it may therefore lie well above or below the observation-side continuation during early and mid follow-up. This is not, by itself, a problem: the inferential object of interest is the blended curve, which closely follows the Kaplan–Meier estimates within the observed window and then transitions smoothly toward the anchored tail, remaining between the observation-side continuation and the external model over 60–120 months.

**Figure 3.**
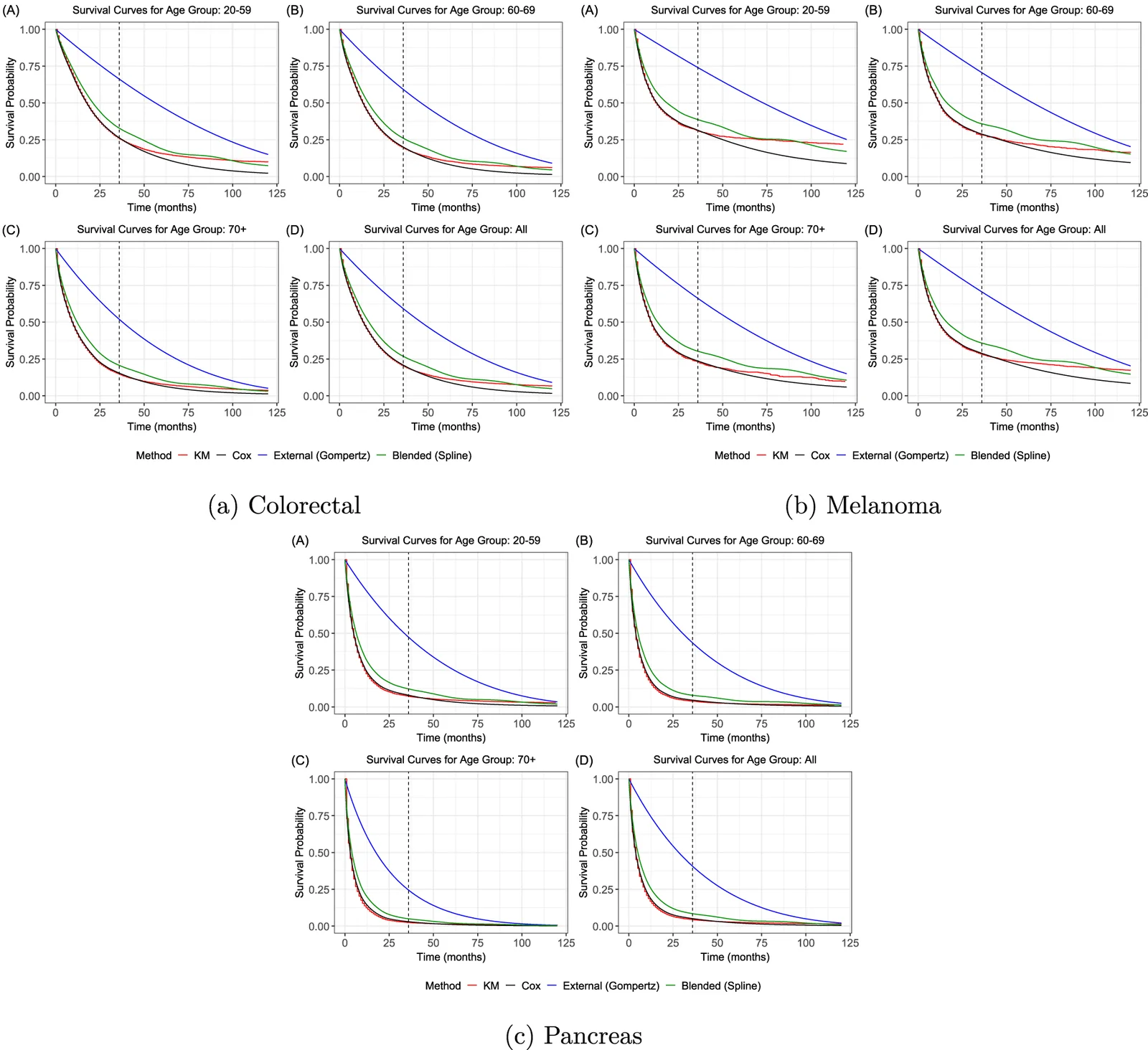
Observed and extrapolated survival curves by cancer type (3-year cutoff; extrapolated to 10 years). The external Gompertz curve is shown as the anchor-driven long-horizon reference component, while inference is based on the blended curve. (a) Colorectal, (b) Melanoma, and (c) Pancreas

Table 6 reports accuracy at 60, 90, and 120 months. Overall performance is stable, and Figure 3 shows that the blended curve tracks Kaplan–Meier estimates within 36 months, then moves smoothly toward the anchored Gompertz tail and stays between the observation-side continuation and the external model over 60–120 months. Pancreatic cancer yields the smallest errors across horizons. Survival absolute errors are ≤0.020 for all age groups, and MAE lies between 0.007 and 0.017 . This pattern is consistent with fast early failure, low long-term survival, and a short extrapolation span. The anchor also aligns well with the observation-side continuation, which reduces mid-horizon drift. Colorectal cancer shows small and homogeneous errors across age groups. MAE ranges from 0.021 to 0.027 , and survival AE is ≤0.031 . The 70+ group attains the lowest 120-month survival AE at 0.007 , indicating that the weight function transfers more mass to the tail when hazards grow with age. Melanoma has the largest errors among the three cancers, driven mainly by RMST at 90 months in older groups. For ages 60–69 and 70+, the RMST AE at 90 months reaches 2.443 and 2.398, while the 120-month survival AE is as low as 0.010 – 0.012 . The mid-horizon curvature is harder to match, and censoring is higher, so the spline weight adjusts more in the 60–90 month band. Even so, long-horizon survival remains well controlled. Age patterns are clear in colorectal and pancreas, where older groups tend to have smaller long-horizon survival errors. Melanoma shows a weaker age gradient, which suggests stronger heterogeneity in the tail.

**Table 6.** SEER extrapolation accuracy at 60/90/120 months.

		AE (Surv)			AE (RMST)			
Cancer	Age group	60	90	120	60	90	120	MAE
Colorectal	20–59	0.031	0.013	0.026	1.071	1.337	0.248	0.027
	60–69	0.029	0.014	0.016	0.987	1.302	0.197	0.025
	70+	0.026	0.013	0.007	0.879	1.166	0.541	0.021
	All	0.030	0.012	0.019	1.002	1.249	0.035	0.025
Melanoma	20–59	0.025	0.003	0.049	1.038	0.958	1.563	0.029
	60–69	0.047	0.038	0.012	1.399	2.443	1.042	0.038
	70+	0.046	0.038	0.010	1.370	2.398	2.092	0.039
	All	0.041	0.022	0.027	1.303	1.828	0.001	0.034
Pancreas	20–59	0.017	0.008	0.009	0.641	0.809	0.178	0.015
	60–69	0.020	0.015	0.001	0.635	1.038	0.804	0.017
	70+	0.008	0.004	0.001	0.320	0.420	0.252	0.007
	All	0.017	0.009	0.003	0.575	0.801	0.437	0.014

AE(Surv): absolute error of survival probability. AE(RMST): absolute error of restricted mean survival time (months). All metrics are computed at the indicated horizons using the spline-blended method.

Methodologically, the external curve is best viewed as a long-horizon reference component rather than the final extrapolated estimate. The blended curve stays between the observation-side continuation and the external curve, which avoids over-commitment to either source while still allowing the external anchor to influence longer-term behavior. A high anchor or a low anchor can shift mid-horizon RMST, but the 120-month survival AE is usually stable. For practice, we recommend stress tests that vary the anchor and focus on 60–90 months for RMST and on 120 months for survival. If a cohort has very poor prognosis, the long-horizon extrapolation is typically more stable and may require fewer sensitivity runs.

### 4.3. Sensitivity to external information in the SEER application

Because the blended approaches explicitly use externally specified long-horizon survival targets, it is important to assess how sensitive the real-data results are to these inputs. We therefore conducted an additional sensitivity analysis in the SEER application by varying the externally specified 120-month survival target for each disease-age cohort under three clinically plausible scenarios: *Base*, *Low*, and *High*. Here, the *Base* scenario corresponds to the 120-month external survival target used in the main analysis, while the *Low* and *High* scenarios were defined by decreasing or increasing that target by 20%, respectively. Thus, for each cohort, if the main-analysis anchor is denoted by $S_{120}^{\mathrm{base}}$ , we set $S_{120}^{\mathrm{low}}=0.8\;S_{120}^{\mathrm{base}}$ and $S_{120}^{\mathrm{high}}=1.2\;S_{120}^{\mathrm{base}}$ . The observation-side model, the 36-month cutoff, and all other modeling choices were kept unchanged. Table 7 summarizes the extrapolation-window mean absolute error (MAE) for the adaptive spline-blended method and the fixed-schedule Beta method under the three external-information scenarios, together with the best-performing fixed parametric comparator for each cohort. Because the fixed parametric models do not use external information, their performance does not vary across the Low/Base/High settings; for conciseness, we therefore report only the best fixed parametric benchmark in the main text. Full results for all metrics and all parametric comparators are reported in Supplementary Tables S2A–S2C.

**Table 7.** Sensitivity of SEER extrapolation performance to external 120-month survival targets.

				Blended (Spline): MAE			Blended (Beta): MAE		
Disease	Age group	Best fixed parametric	Best fixed MAE	Low	Base	High	Low	Base	High
Colorectal	20–59	Log-logistic	0.016	0.026	0.027	0.030	0.025	0.038	0.051
Colorectal	60–69	Log-logistic	0.007	0.023	0.025	0.027	0.018	0.028	0.037
Colorectal	70+	Log-logistic	0.003	0.019	0.021	0.023	0.014	0.019	0.025
Colorectal	All	Log-logistic	0.007	0.023	0.025	0.027	0.015	0.025	0.034
Melanoma	20–59	Log-normal	0.097	0.035	0.029	0.031	0.016	0.036	0.055
Melanoma	60–69	Log-logistic	0.068	0.033	0.038	0.047	0.023	0.042	0.062
Melanoma	70+	Log-logistic	0.046	0.030	0.039	0.047	0.031	0.045	0.059
Melanoma	All	Log-logistic	0.078	0.032	0.034	0.039	0.016	0.034	0.053
Pancreas	20–59	Log-logistic	0.019	0.014	0.015	0.016	0.009	0.013	0.017
Pancreas	60–69	Log-logistic	0.008	0.015	0.017	0.019	0.013	0.017	0.020
Pancreas	70+	Log-logistic	0.004	0.006	0.007	0.008	0.004	0.005	0.005
Pancreas	All	Log-logistic	0.010	0.013	0.014	0.014	0.010	0.012	0.014

Notes: Low, Base, and High denote three clinically plausible external-information scenarios defined through alternative 120-month survival targets for each disease-age cohort. MAE is computed over the extrapolation window beyond the 36-month observation cutoff. For fixed parametric models, performance does not vary across Low/Base/High because they do not use external information; the table therefore reports only the best fixed parametric comparator, defined as the model with the smallest MAE among the Exponential, Weibull, Log-normal, and Log-logistic fits for that cohort. Full results for all models and all metrics are reported in Supplementary Tables S2A–S2C.

First, the real-data results do depend on the externally supplied long-horizon target, confirming that the quality of external information matters in practice. Second, this dependence is generally stronger for the fixed-schedule Beta method than for the adaptive spline method, especially in cohorts with more substantial long-horizon uncertainty. For example, in the colorectal all-age cohort, the Beta MAE increases from 0.015 under the Low scenario to 0.034 under the High scenario, whereas the spline MAE changes more modestly from 0.023 to 0.027 . A similar pattern appears in the melanoma all-age cohort, where the Beta MAE rises from 0.016 to 0.053 , while the spline MAE changes from 0.032 to 0.039 . These results are consistent with the methodological difference between the two approaches: the Beta method is forced to hand over fully to the external tail by the horizon, whereas the spline method allows adaptive partial borrowing and is therefore less tightly tied to any one externally specified endpoint. At the same time, the results also show that the adaptive spline approach is not uniformly optimal in every cohort. In several pancreatic cohorts with very poor prognosis, both blended approaches are relatively stable and the Beta method is slightly more accurate on MAE. More broadly, some standard parametric models remain strong competitors in selected cohorts, particularly the log-logistic model and, in one melanoma subgroup, the log-normal model. We therefore do not interpret the SEER sensitivity analysis as showing universal dominance of the spline approach. Rather, the added value of the adaptive spline method is that it often provides a more robust extrapolation profile when the external long-horizon input is informative but not known to match the truth exactly. This point is important in applied HTA. The fixed-schedule Beta method can perform very well when the externally specified long-horizon survival target happens to align closely with the later observed survival pattern. However, such alignment cannot usually be verified at the time extrapolation is performed. The practical motivation for the adaptive spline approach is precisely to reduce reliance on that exact-match condition, while still allowing clinically interpretable external information to shape the long-term tail.

## 5. Conclusion

Extrapolating survival beyond observed follow-up is unavoidable in HTA and many clinical studies, yet long-horizon accuracy remains sensitive to model misspecification and to how external information is incorporated.^3^ This paper proposed an adaptive spline-blended framework that combines a short-term, data-driven observation-side model with an identifiable anchored Gompertz tail on the cumulative-hazard scale. The key innovation is a monotone P-spline mapping of the contrast $z(t)=\log\;H_{\mathrm{obs}}(t)-\log\;H_{\mathrm{ext}}(t)$ into a time-varying weight π(t) , coupled with a simple temperature parameter that controls the slope of $\pi^{\prime }(t)$ and guarantees nonnegativity of the blended hazard on a chosen grid. The construction is modular, interpretable (via the identity in (16)), and readily implementable with standard tools for survival modeling, shape-constrained smoothing, and approximate Bayesian inference.^7,11,13^

Across extensive simulations, the adaptive blend achieved consistently lower or competitive errors relative to fixed-schedule blending and single-family parametric models, particularly at longer horizons where rigid schedules and global parametrics are most vulnerable.^26,27^ In a large SEER application, the method tracked Kaplan–Meier estimates closely within the observed window and then transitioned smoothly toward the anchored external tail, yielding small long-horizon survival errors while respecting clinical plausibility. Using a Gompertz tail gives a transparent parameterization for exponentially bending hazards and connects to classical mortality modeling,^12^ while anchor-based identification lets external knowledge enter as prior information without introducing outcomes beyond the cutoff.^28,29^ Relative to the fixed-schedule Beta approach, the adaptive spline method is most useful when external long-term information is viewed as informative but potentially imperfect, so that partial borrowing at the horizon may be preferable to a pre-specified full handover. By contrast, the Beta approach remains attractive when analysts want a fully transparent and explicitly scheduled transition to the external tail. We therefore view the two blending strategies as complementary rather than competing defaults, with the appropriate choice depending on the intended role of the external input. More broadly, our method should also be interpreted in the context of other approaches that incorporate external evidence for extrapolation. In particular, recent Bayesian frameworks such as survextrap provide a more general evidence-synthesis route in which individual-level survival data and external aggregate survival information are modeled jointly within a flexible spline-based hazard model.^22^ Our contribution is different in emphasis: we focus on transparent blending between an observation-side component and an anchor-identified tail on the cumulative-hazard scale, with adaptive monotone weighting and a practical temperature-based guarantee of hazard validity. In this sense, our framework is not intended to replace broader joint Bayesian evidence-synthesis models, but rather to provide a comparatively simple, interpretable, and operationally convenient alternative when the analyst wishes to preserve short-term fit while adaptively calibrating long-term behavior.

At the same time, the HTA estimand itself deserves careful attention. Our framework naturally takes the trial-based short-term survival trajectory as the primary starting point and then constrains the long-term tail using external input. In many HTA settings this is a reasonable strategy, but it does not automatically imply that the resulting survival curve is the exact population quantity most relevant for decision-making. If the trial population is materially fitter than the target real-world population, then a method that preserves trial-based behavior too strongly may overstate long-term survival in practice. For that reason, our approach is best suited to settings in which trial follow-up remains the main source of short-term evidence, but longer-term behavior is uncertain and requires external constraint or calibration. When there is substantial mismatch between the trial and target populations, blending alone may be insufficient, and additional population-adjustment or broader evidence-synthesis methods may be needed.

The suitability of external information for the proposed method depends on clinical relevance and temporal transportability rather than on recency alone. More recent external data may be preferable when survival patterns are changing because of evolving treatment standards, screening practices, stage migration, or supportive care, but recency by itself does not guarantee suitability. Conversely, older external information may still be useful if the target population, endpoint definition, and disease context remain sufficiently stable. In practice, users should assess whether the external source is aligned with the target population and estimand, whether major secular changes in survival are likely, whether the implied long-horizon survival level is broadly consistent with the observed data and clinical expectations, and whether conclusions remain robust under sensitivity analyses over plausible alternative anchor values. If the external source provides a full historical survival curve, a short-term alignment check is also helpful: users can compare short-term survival in the external and trial cohorts over the observed follow-up period. For example, if the trial cohort already shows substantially better survival by year 3 than the historical cohort at the same time point, then a historical 10-year anchor may not be directly transportable and may need adjustment or more cautious use before being imposed on the extrapolation. If materially different conclusions arise across clinically reasonable external inputs, then the external information should be treated as weak and the resulting long-horizon extrapolations interpreted more cautiously. A practical rule is that long-horizon external information should not be borrowed uncritically when the external and trial cohorts already show clear misalignment within the observed short-term follow-up period.

The approach also has limitations and clear avenues for future work. Performance depends on the quality and placement of anchors, so sensitivity analysis over $S_{a}$ and $t_{a}$ remains essential. While our sufficient condition for nonnegativity is easy to check and enforce, a fully probabilistic treatment of τ could propagate additional uncertainty. Methodologically, the framework could be extended to multiple anchors, alternative tail families, competing risks, comparative or multi-arm extrapolation, or covariate-dependent weights, and embedded more fully in joint Bayesian inference (e.g., via INLA) to pool uncertainty across components. Beyond oncology, the same ideas may be useful in other disease areas where follow-up is limited but long-term extrapolation is important, provided that clinically interpretable external information is available. Practically, the method integrates with widely used software (flexsurv for parametrics, scam/mgcv for monotone smoothing, and INLA for fast latent-Gaussian computation), lowering the barrier to routine use in HTA workflows.^3,7,11,13^

From a practical perspective, the method is most useful when the short-term trial follow-up is reasonably well captured by the observation-side model, longer-term survival remains uncertain, and some clinically interpretable external information is available to anchor the tail. By contrast, caution is needed when the short-term fit is poor, when the external anchor is weakly justified or highly outdated, or when the trial population differs materially from the target population for decision-making. In applied work, we recommend checking the adequacy of the observation-side fit, varying the anchor level and anchor time in sensitivity analyses, and inspecting the resulting blended curve for clinical plausibility. In comparative settings with active and control arms, the framework can in principle be applied separately to each arm, but the resulting long-term treatment-effect behavior should then be examined carefully to avoid implausible crossing, divergence, or convergence patterns. Further practical notes for users are provided in Appendix C, which complements the package documentation distributed with survblendr.”

In summary, blending on the cumulative-hazard scale with an adaptive, shape-constrained weight and an anchored external tail provides a robust, transparent, and implementable route to survival extrapolation. It preserves short-term fidelity, allows adaptive borrowing from external long-term information, and offers explicit guarantees for hazard validity—features that are especially useful when long-horizon inputs are informative but not taken to be exact.

## Supplemental Material

sj-pdf-1-smm-10.1177_09622802261458072 - Supplemental material for Adaptive spline-based weighting functions for blended survival curve extrapolation Supplemental material, sj-pdf-1-smm-10.1177_09622802261458072 for Adaptive spline-based weighting functions for blended survival curve extrapolation by Hao Chen and Clara Grazian in Statistical Methods in Medical Research

## Data Availability

The SEER cancer registry data used in this study is available from the Surveillance, Epidemiology, and End Results (SEER) Program (www.seer.cancer.gov) upon request. An open-source implementation is available as the R package survblendr, with source code at https://github.com/haohaostats/survblendr
