# Supplementary material for: Adaptive spline-based weighting functions for blended survival curve extrapolation
Source: Stat Methods Med Res. 2026 Jul 16;35(8):1600–19. doi: 10.1177/09622802261458072 (PMC13719905; doi:10.1177/09622802261458072)
Supplement: Supplementary file 1 [file sj-pdf-1-smm-10.1177_09622802261458072.pdf]

Supplementary Material for

Adaptive Spline-Based Weighting Functions for Blended Survival Curve Extrapolation

This supplementary material contains the mathematical derivations, practical notes, and additional simulation and SEER sensitivity tables accompanying the main article. Equations numbered as (S1), (S2), etc. are equations in this Supplementary Material. Equation numbers shown without an "S" prefix, such as main-text equation (1), refer to the corresponding numbered equations in the main article.

A Cumulative-Hazard Blending: Identities and Proofs

Proposition A.1 (Equivalence of log–survival and cumulative–hazard blending)**.** *Let $S_{\text{obs}}(t)$ and $S_{\text{ext}}(t)$ be valid survival functions on $[0, T_\star]$ and let $\pi(t) \in [0, 1]$. Then the log–survival blending in (1) is equivalent to the cumulative–hazard blending in (2). Moreover,*

$$S_{\text{blend}}(t) = \exp\{(1 - \pi(t)) \log S_{\text{obs}}(t) + \pi(t) \log S_{\text{ext}}(t)\} = S_{\text{obs}}(t)^{\,1-\pi(t)}\, S_{\text{ext}}(t)^{\,\pi(t)} \in (0, 1].$$

Proof. By definition $H(t) = -\log S(t)$, hence $\log S(t) = -H(t)$. Substituting into (1) yields

$$\log S_{\text{blend}}(t) = -(1 - \pi(t))H_{\text{obs}}(t) - \pi(t)H_{\text{ext}}(t),$$

so taking negatives gives (2). Exponentiating (1) yields the geometric mixture form. Since $S_{\text{obs}}, S_{\text{ext}} \in (0, 1]$ and $0 \le \pi \le 1$, the product lies in $(0, 1]$. □

Proposition A.2 (Hazard under time–varying weight)**.** *Assume $S_{\text{obs}}, S_{\text{ext}}$ are differentiable and π is differentiable on $(0, T_\star)$. Then the blended hazard equals*

$$h_{\text{blend}}(t) = (1 - \pi(t))\, h_{\text{obs}}(t) + \pi(t)\, h_{\text{ext}}(t) + \{H_{\text{ext}}(t) - H_{\text{obs}}(t)\}\, \pi'(t),$$

which is (3).

Proof. Differentiate (2) using the product rule:

$$\frac{d}{dt}\big[(1 - \pi)H_{\text{obs}}\big] = -(\pi')H_{\text{obs}} + (1 - \pi)h_{\text{obs}}, \quad \frac{d}{dt}\big[\pi H_{\text{ext}}\big] = (\pi')H_{\text{ext}} + \pi h_{\text{ext}}.$$

Summing gives the stated expression. □

Corollary A.3 (Constant weight)**.** *If $\pi'(t) \equiv 0$, then $h_{\text{blend}}(t) = (1 - \pi)h_{\text{obs}}(t) + \pi h_{\text{ext}}(t)$ is the pointwise convex combination of hazards.*

Remark A.4 (Interpretability identity). Whenever $H_{\text{ext}}(t) \neq H_{\text{obs}}(t)$,

$$\pi(t) = \frac{H_{\text{blend}}(t) - H_{\text{obs}}(t)}{H_{\text{ext}}(t) - H_{\text{obs}}(t)},$$

so the weight equals the relative position of H_{blend} between H_{obs} and H_{ext} on the cumulative–hazard scale.

Lemma A.5 (Hazard nonnegativity under temperature scaling)**.** *Let $\eta(t)$ be monotone increasing on $[0, T_\star]$ and define $\pi_\tau(t) = \{1+\exp[-\eta(t)/\tau]\}^{-1}$ with $\tau \geq 1$. For any grid $\{t_g\}_{g=1}^{G} \subset [0, T_\star]$, if*

$$\pi'_\tau(t_g) \;\leq\; \frac{(1 - \pi_\tau(t_g))\,\widehat{h}_{\text{obs}}(t_g) + \pi_\tau(t_g)\,\widehat{h}_{\text{ext}}(t_g)}{|\widehat{H}_{\text{ext}}(t_g) - \widehat{H}_{\text{obs}}(t_g)|} \quad \textit{for all } g, \tag{S1}$$

then the blended hazard in (15) satisfies $\widehat{h}_{\text{blend}}(t_g) \geq 0$ for all grid points.

Proof. By (15), $\widehat{h}_{\text{blend}}(t_g) = (1 - \pi_\tau)\widehat{h}_{\text{obs}} + \pi_\tau \widehat{h}_{\text{ext}} + \{\widehat{H}_{\text{ext}} - \widehat{H}_{\text{obs}}\}\pi'_\tau$. Condition (S1) ensures the drift term cannot drive the sum below 0. □

Corollary A.6 (Existence and minimal τ selection)**.** *Since $\sigma(u) = \{1+\exp(-u)\}^{-1}$ has derivative $\sigma'(u) = \sigma(u)\{1 - \sigma(u)\} \leq \frac{1}{4}$ for all u,*

$$\pi'_\tau(t) = \tfrac{1}{\tau}\,\sigma'\{\eta(t)/\tau\}\,\eta'(t) \;\leq\; \frac{1}{4\tau}\,\eta'(t). \tag{S2}$$

Hence there exists a sufficiently large τ so that (S1) holds on the grid. In implementation we choose the smallest $\tau \geq 1$ *satisfying (S1) for all t_g, which guarantees nonnegativity while preserving maximal adaptivity.*

B Proofs for the anchored Gompertz tail

This appendix details the mathematical properties of the anchored Gompertz tail used in our framework. The general properties of the Gompertz distribution are well established in the survival-analysis literature; see, [1–4]. We include brief proofs of identifiability, positivity, and the exponential limit under our anchor-based parameterization solely for completeness.

Recall the external tail (Section 2.4.2 of the main article):

$$h_{\text{ext}}(t) = \rho e^{\gamma t}, \qquad H_{\text{ext}}(t) = \frac{\rho}{\gamma}(e^{\gamma t} - 1), \qquad S_{\text{ext}}(t) = \exp\Big\{-\tfrac{\rho}{\gamma}(e^{\gamma t} - 1)\Big\}, \tag{7}$$

and the anchor condition at $t_a \in [t_{\text{obs}}, T_\star]$:

$$S_{\text{ext}}(t_a) = S_a \in (0, 1). \tag{8}$$

Define, for $\gamma \neq 0$,

$$\rho(\gamma, S_a) = \gamma\,\frac{-\log S_a}{e^{\gamma t_a} - 1}. \tag{9}$$

B.1 Identifiability under the anchor

Claim. For fixed $t_a \in [t_{\text{obs}}, T_\star]$ and $S_a \in (0,1)$, the parameterization $(\gamma, S_a) \mapsto (\rho(\gamma, S_a), \gamma)$ yields an identifiable anchored Gompertz tail; moreover, it extends continuously to $\gamma = 0$.

Proof. By (7)–(8),

$$-\log S_a = \frac{\rho}{\gamma}(e^{\gamma t_a} - 1).$$

For any $\gamma \neq 0$, the coefficient $(e^{\gamma t_a} - 1)/\gamma \neq 0$, hence

$$\rho = \rho(\gamma, S_a) = \gamma \, \frac{-\log S_a}{e^{\gamma t_a} - 1}$$

is the unique value satisfying the anchor. Conversely, given (ρ, γ) with $\gamma \neq 0$, the anchor determines $S_a = \exp\{-\frac{\rho}{\gamma}(e^{\gamma t_a} - 1)\} \in (0,1)$ uniquely. Therefore the tail is identifiable under (γ, S_a) for $\gamma \neq 0$. For $\gamma \to 0$, using $e^{\gamma t_a} - 1 = \gamma t_a + o(\gamma)$,

$$\lim_{\gamma \to 0} \rho(\gamma, S_a) = \lim_{\gamma \to 0} \gamma \, \frac{-\log S_a}{\gamma t_a + o(\gamma)} = \frac{-\log S_a}{t_a},$$

so the mapping extends continuously at $\gamma = 0$. □

B.2 Positivity of $\rho(\gamma, S_a)$

Claim. For any $S_a \in (0,1)$ and any $\gamma \in \mathbb{R}$ (including the limit $\gamma = 0$), one has $\rho(\gamma, S_a) > 0$.

Proof. Since $S_a \in (0,1)$, we have $-\log S_a > 0$. If $\gamma > 0$, then $e^{\gamma t_a} - 1 > 0$, hence $\rho(\gamma, S_a) > 0$. If $\gamma < 0$, then $e^{\gamma t_a} - 1 \in (-1, 0)$ and the numerator $\gamma(-\log S_a) < 0$, so the ratio is again positive. For $\gamma = 0$ we use the limit in Subsection B.1: $\rho(0, S_a) = (-\log S_a)/t_a > 0$. □

B.3 Exponential-limit continuity at $\gamma = 0$

Claim. As $\gamma \to 0$, the anchored Gompertz tail converges to the anchored exponential tail with rate $\lambda = (-\log S_a)/t_a$:

$$S_{\text{ext}}(t) \longrightarrow \exp(-\lambda t), \qquad h_{\text{ext}}(t) \longrightarrow \lambda.$$

Proof. From Subsection B.1, $\rho(\gamma, S_a) \to \lambda$. Using $e^{\gamma t} - 1 = \gamma t + o(\gamma)$,

$$\frac{\rho(\gamma, S_a)}{\gamma}(e^{\gamma t} - 1) \;=\; \frac{\rho(\gamma, S_a)}{\gamma}\, \gamma t \,[1 + o(1)] \;\longrightarrow\; \lambda t,$$

hence $S_{\text{ext}}(t) = \exp\{-[\rho(\gamma, S_a)/\gamma](e^{\gamma t} - 1)\} \to e^{-\lambda t}$; differentiating gives $h_{\text{ext}}(t) \to \lambda$. □

B.4 Monotonicity of the hazard and doubling time

Claim. Under the Gompertz tail, $h_{\text{ext}}(t) = \rho e^{\gamma t}$ is increasing in t if $\gamma > 0$, decreasing if $\gamma < 0$, and constant if $\gamma = 0$. The hazard–doubling time equals

$$T_{\text{double}} = \frac{\ln 2}{\gamma} \quad (\gamma > 0).$$

Proof. Immediate from $\partial h_{\text{ext}}(t)/\partial t = \gamma h_{\text{ext}}(t)$. For the doubling time, solve $h_{\text{ext}}(t + T)/h_{\text{ext}}(t) = e^{\gamma T} = 2$, giving $T = (\ln 2)/\gamma$. $\square$

C Practical notes for users of `survblendr`

This appendix provides brief practical guidance for readers who may wish to apply the adaptive spline–blended extrapolation framework in real analyses. It is intended as a concise in-paper note rather than a full software manual. A separate user-oriented handbook, ***survblendr*****: *A Short Practical Guide***, together with worked examples and package documentation, is available in the GitHub repository:

https://github.com/haohaostats/survblendr

C.1 Suitable use contexts

The method is most useful when the short-term follow-up period is reasonably well captured by the observation-side model, longer-term survival remains uncertain, and some clinically interpretable external information is available to anchor the long-term tail. In such settings, the framework is especially attractive when the external input is regarded as informative but not exact, so that adaptive partial borrowing is preferable to a forced full handover at the extrapolation horizon.

C.2 Situations requiring caution

Caution is needed when the observation-side model fits the observed follow-up poorly, when the external anchor is weakly justified or outdated, or when the trial population differs materially from the target population for decision-making. In such cases, blending alone may be insufficient, and broader evidence-synthesis or population-adjustment strategies may be needed.

C.3 Recommended checks

In applied work, we recommend verifying the adequacy of the observation-side fit over the observed period, varying the anchor level S_a and anchor time t_a in sensitivity analyses, and inspecting the resulting blended curve for clinical plausibility. In many practical settings, the robustness of long-horizon conclusions to reasonable anchor variation is as important as the primary extrapolated estimate itself.

C.4 Comparative settings

In comparative settings, the framework can in principle be applied separately to active and control arms. However, users should then examine the implied long-term treatment-effect behaviour carefully, since arm-specific extrapolations that appear reasonable in isolation may still generate implausible crossing, excessive divergence, or unrealistic convergence when considered jointly. Extending the present framework to richer comparative or multi-arm settings remains an important direction for future work.

Appendix Table A1. Summary of simulation settings across Scenarios 1–3.

**Scenario**	**Purpose**	**Data-generating model(s)**	t_{obs}	$T_\star$	**Anchor mean** S_a	**Censoring**	**Notes**
1	Main comparison across censoring levels	Weibull	10	20	0.10	10%, 20%, 30%, 40%, 50%	True $S(20) = 0.20$
2	Main comparison across non-Weibull truths	Exponential, Log-normal, Log-logistic	10	20	0.10	50%	True $S(20) = 0.20$
3	Sensitivity analysis	Weibull	8, 10, 12	20	0.10, 0.12, 0.14, 0.16, 0.18, 0.20	30%	Full grid over t_{obs} and S_a

Supplementary Table S1A. Full Scenario 3 results for $t_{\text{obs}} = 8$ years.

S_a	Model	AE (Surv) 15y		AE (Surv) 20y		AE (RMST) 15y		AE (RMST) 20y		MAE		FD	
		Mean	SD	Mean	SD	Mean	SD	Mean	SD	Mean	SD	Mean	SD
0.10	Blended (Spline)	0.013	0.009	0.011	0.008	0.082	0.060	0.130	0.095	0.012	0.007	0.050	0.038
	Blended (Beta)	0.043	0.007	0.100	0.001	0.095	0.061	0.731	0.052	0.053	0.003	0.637	0.022
	Weibull	0.030	0.019	0.028	0.020	0.198	0.114	0.330	0.216	0.029	0.017	0.137	0.105
	Exponential	0.184	0.013	0.247	0.014	1.002	0.084	2.371	0.157	0.166	0.012	1.369	0.073
	Lognormal	0.150	0.017	0.213	0.020	0.807	0.101	2.006	0.203	0.137	0.015	1.198	0.105
	Loglogistic	0.090	0.018	0.133	0.020	0.492	0.106	1.247	0.206	0.084	0.016	0.754	0.103
0.12	Blended (Spline)	0.015	0.011	0.014	0.010	0.081	0.060	0.145	0.108	0.013	0.008	0.066	0.049
	Blended (Beta)	0.021	0.007	0.080	0.001	0.057	0.042	0.531	0.053	0.042	0.005	0.475	0.073
	Weibull	0.030	0.019	0.028	0.020	0.198	0.114	0.330	0.216	0.029	0.017	0.137	0.105
	Exponential	0.184	0.013	0.247	0.014	1.002	0.084	2.371	0.157	0.166	0.012	1.369	0.073
	Lognormal	0.150	0.017	0.213	0.020	0.807	0.101	2.006	0.203	0.137	0.015	1.198	0.105
	Loglogistic	0.090	0.018	0.133	0.020	0.492	0.106	1.247	0.206	0.084	0.016	0.754	0.103
0.14	Blended (Spline)	0.024	0.014	0.021	0.012	0.127	0.080	0.231	0.140	0.019	0.011	0.104	0.062
	Blended (Beta)	0.007	0.005	0.060	0.001	0.105	0.065	0.334	0.058	0.033	0.006	0.232	0.115
	Weibull	0.030	0.019	0.028	0.020	0.198	0.114	0.330	0.216	0.029	0.017	0.137	0.105
	Exponential	0.184	0.013	0.247	0.014	1.002	0.084	2.371	0.157	0.166	0.012	1.369	0.073
	Lognormal	0.150	0.017	0.213	0.020	0.807	0.101	2.006	0.203	0.137	0.015	1.198	0.105
	Loglogistic	0.090	0.018	0.133	0.020	0.492	0.106	1.247	0.206	0.084	0.016	0.754	0.103
0.16	Blended (Spline)	0.034	0.014	0.031	0.013	0.192	0.085	0.344	0.148	0.029	0.012	0.152	0.064
	Blended (Beta)	0.017	0.008	0.040	0.001	0.179	0.074	0.145	0.056	0.028	0.008	0.107	0.080
	Weibull	0.030	0.019	0.028	0.020	0.198	0.114	0.330	0.216	0.029	0.017	0.137	0.105
	Exponential	0.184	0.013	0.247	0.014	1.002	0.084	2.371	0.157	0.166	0.012	1.369	0.073
	Lognormal	0.150	0.017	0.213	0.020	0.807	0.101	2.006	0.203	0.137	0.015	1.198	0.105
	Loglogistic	0.090	0.018	0.133	0.020	0.492	0.106	1.247	0.206	0.084	0.016	0.754	0.103
0.18	Blended (Spline)	0.045	0.014	0.041	0.013	0.263	0.084	0.466	0.146	0.039	0.012	0.203	0.063
	Blended (Beta)	0.034	0.009	0.020	0.002	0.253	0.076	0.057	0.043	0.028	0.008	0.197	0.049
	Weibull	0.030	0.019	0.028	0.020	0.198	0.114	0.330	0.216	0.029	0.017	0.137	0.105
	Exponential	0.184	0.013	0.247	0.014	1.002	0.084	2.371	0.157	0.166	0.012	1.369	0.073
	Lognormal	0.150	0.017	0.213	0.020	0.807	0.101	2.006	0.203	0.137	0.015	1.198	0.105
	Loglogistic	0.090	0.018	0.133	0.020	0.492	0.106	1.247	0.206	0.084	0.016	0.754	0.103
0.20	Blended (Spline)	0.056	0.014	0.051	0.013	0.333	0.080	0.586	0.141	0.049	0.012	0.253	0.062
	Blended (Beta)	0.050	0.009	0.001	0.001	0.322	0.077	0.224	0.056	0.035	0.009	0.098	0.024
	Weibull	0.030	0.019	0.028	0.020	0.198	0.114	0.330	0.216	0.029	0.017	0.137	0.105
	Exponential	0.184	0.013	0.247	0.014	1.002	0.084	2.371	0.157	0.166	0.012	1.369	0.073
	Lognormal	0.150	0.017	0.213	0.020	0.807	0.101	2.006	0.203	0.137	0.015	1.198	0.105
	Loglogistic	0.090	0.018	0.133	0.020	0.492	0.106	1.247	0.206	0.084	0.016	0.754	0.103

Supplementary Table S1B. Full Scenario 3 results for $t_{\text{obs}} = 10$ years.

S_a	Model	AE (Surv) 15y		AE (Surv) 20y		AE (RMST) 15y		AE (RMST) 20y		MAE		FD	
		Mean	SD	Mean	SD	Mean	SD	Mean	SD	Mean	SD	Mean	SD
0.10	Blended (Spline)	0.012	0.009	0.013	0.009	0.058	0.042	0.125	0.086	0.012	0.008	0.068	0.044
	Blended (Beta)	0.025	0.008	0.100	0.001	0.038	0.029	0.605	0.037	0.055	0.003	0.567	0.048
	Weibull	0.029	0.016	0.026	0.017	0.143	0.069	0.265	0.161	0.028	0.014	0.125	0.082
	Exponential	0.144	0.011	0.204	0.011	0.582	0.051	1.669	0.107	0.146	0.011	1.088	0.061
	Lognormal	0.116	0.014	0.173	0.016	0.461	0.062	1.391	0.146	0.119	0.014	0.929	0.082
	Loglogistic	0.075	0.013	0.116	0.015	0.306	0.061	0.933	0.137	0.079	0.013	0.627	0.075
0.12	Blended (Spline)	0.010	0.007	0.009	0.007	0.040	0.030	0.086	0.062	0.009	0.006	0.047	0.035
	Blended (Beta)	0.010	0.007	0.080	0.001	0.058	0.040	0.446	0.036	0.043	0.003	0.388	0.074
	Weibull	0.026	0.016	0.022	0.017	0.129	0.069	0.233	0.161	0.025	0.015	0.109	0.088
	Exponential	0.142	0.011	0.202	0.011	0.572	0.051	1.648	0.107	0.144	0.011	1.076	0.057
	Lognormal	0.113	0.014	0.171	0.016	0.451	0.062	1.369	0.146	0.116	0.014	0.918	0.086
	Loglogistic	0.073	0.013	0.114	0.015	0.294	0.061	0.909	0.137	0.076	0.013	0.615	0.077
0.14	Blended (Spline)	0.014	0.010	0.010	0.008	0.062	0.042	0.108	0.082	0.012	0.009	0.048	0.040
	Blended (Beta)	0.009	0.007	0.060	0.001	0.099	0.050	0.285	0.036	0.034	0.005	0.186	0.085
	Weibull	0.026	0.016	0.022	0.017	0.129	0.069	0.233	0.161	0.025	0.015	0.109	0.088
	Exponential	0.142	0.011	0.202	0.011	0.572	0.051	1.648	0.107	0.144	0.011	1.076	0.057
	Lognormal	0.113	0.014	0.171	0.016	0.451	0.062	1.369	0.146	0.116	0.014	0.918	0.086
	Loglogistic	0.073	0.013	0.114	0.015	0.294	0.061	0.909	0.137	0.076	0.013	0.615	0.077
0.16	Blended (Spline)	0.025	0.011	0.018	0.011	0.113	0.047	0.197	0.102	0.022	0.010	0.084	0.055
	Blended (Beta)	0.022	0.010	0.040	0.001	0.143	0.052	0.126	0.041	0.027	0.006	0.074	0.058
	Weibull	0.026	0.016	0.022	0.017	0.129	0.069	0.233	0.161	0.025	0.015	0.109	0.088
	Exponential	0.142	0.011	0.202	0.011	0.572	0.051	1.648	0.107	0.144	0.011	1.076	0.057
	Lognormal	0.113	0.014	0.171	0.016	0.451	0.062	1.369	0.146	0.116	0.014	0.918	0.086
	Loglogistic	0.073	0.013	0.114	0.015	0.294	0.061	0.909	0.137	0.076	0.013	0.615	0.077
0.18	Blended (Spline)	0.036	0.011	0.028	0.011	0.166	0.046	0.300	0.100	0.033	0.010	0.134	0.055
	Blended (Beta)	0.034	0.010	0.020	0.001	0.182	0.053	0.040	0.028	0.026	0.007	0.142	0.038
	Weibull	0.026	0.016	0.022	0.017	0.129	0.069	0.233	0.161	0.025	0.015	0.109	0.088
	Exponential	0.142	0.011	0.202	0.011	0.572	0.051	1.648	0.107	0.144	0.011	1.076	0.057
	Lognormal	0.113	0.014	0.171	0.016	0.451	0.062	1.369	0.146	0.116	0.014	0.918	0.086
	Loglogistic	0.073	0.013	0.114	0.015	0.294	0.061	0.909	0.137	0.076	0.013	0.615	0.077
0.20	Blended (Spline)	0.047	0.011	0.038	0.011	0.219	0.044	0.403	0.098	0.043	0.010	0.185	0.055
	Blended (Beta)	0.046	0.010	0.001	0.001	0.218	0.054	0.178	0.041	0.032	0.007	0.040	0.017
	Weibull	0.026	0.016	0.022	0.017	0.129	0.069	0.233	0.161	0.025	0.015	0.109	0.088
	Exponential	0.142	0.011	0.202	0.011	0.572	0.051	1.648	0.107	0.144	0.011	1.076	0.057
	Lognormal	0.113	0.014	0.171	0.016	0.451	0.062	1.369	0.146	0.116	0.014	0.918	0.086
	Loglogistic	0.073	0.013	0.114	0.015	0.294	0.061	0.909	0.137	0.076	0.013	0.615	0.077

Supplementary Table S1C. Full Scenario 3 results for $t_{\text{obs}} = 12$ years.

S_a	Model	AE (Surv) 15y		AE (Surv) 20y		AE (RMST) 15y		AE (RMST) 20y		MAE		FD	
		Mean	SD	Mean	SD	Mean	SD	Mean	SD	Mean	SD	Mean	SD
0.10	Blended (Spline)	0.015	0.009	0.021	0.010	0.042	0.025	0.145	0.078	0.017	0.010	0.103	0.053
	Blended (Beta)	0.009	0.007	0.100	0.001	0.063	0.031	0.483	0.030	0.057	0.002	0.421	0.060
	Weibull	0.028	0.014	0.023	0.015	0.083	0.039	0.199	0.116	0.026	0.014	0.117	0.076
	Exponential	0.111	0.011	0.169	0.011	0.283	0.031	1.137	0.087	0.129	0.011	0.855	0.056
	Lognormal	0.087	0.013	0.141	0.015	0.220	0.038	0.930	0.114	0.104	0.014	0.711	0.077
	Loglogistic	0.060	0.013	0.100	0.013	0.154	0.037	0.655	0.104	0.072	0.013	0.501	0.068
0.12	Blended (Spline)	0.008	0.006	0.012	0.009	0.021	0.016	0.076	0.058	0.009	0.006	0.056	0.046
	Blended (Beta)	0.016	0.009	0.080	0.001	0.080	0.034	0.352	0.032	0.046	0.003	0.273	0.065
	Weibull	0.028	0.014	0.023	0.015	0.083	0.039	0.199	0.116	0.026	0.014	0.117	0.076
	Exponential	0.111	0.011	0.169	0.011	0.283	0.031	1.137	0.087	0.129	0.011	0.855	0.056
	Lognormal	0.087	0.013	0.141	0.015	0.220	0.038	0.930	0.114	0.104	0.014	0.711	0.077
	Loglogistic	0.060	0.013	0.100	0.013	0.154	0.037	0.655	0.104	0.072	0.013	0.501	0.068
0.14	Blended (Spline)	0.012	0.007	0.008	0.007	0.035	0.021	0.069	0.049	0.011	0.006	0.039	0.034
	Blended (Beta)	0.024	0.011	0.060	0.001	0.095	0.035	0.224	0.032	0.036	0.004	0.129	0.066
	Weibull	0.028	0.014	0.023	0.015	0.083	0.039	0.199	0.116	0.026	0.014	0.117	0.076
	Exponential	0.111	0.011	0.169	0.011	0.283	0.031	1.137	0.087	0.129	0.011	0.855	0.056
	Lognormal	0.087	0.013	0.141	0.015	0.220	0.038	0.930	0.114	0.104	0.014	0.711	0.077
	Loglogistic	0.060	0.013	0.100	0.013	0.154	0.037	0.655	0.104	0.072	0.013	0.501	0.068
0.16	Blended (Spline)	0.022	0.009	0.012	0.008	0.066	0.024	0.124	0.069	0.019	0.008	0.062	0.042
	Blended (Beta)	0.032	0.012	0.040	0.001	0.109	0.036	0.101	0.033	0.028	0.005	0.055	0.041
	Weibull	0.028	0.014	0.023	0.015	0.083	0.039	0.199	0.116	0.026	0.014	0.117	0.076
	Exponential	0.111	0.011	0.169	0.011	0.283	0.031	1.137	0.087	0.129	0.011	0.855	0.056
	Lognormal	0.087	0.013	0.141	0.015	0.220	0.038	0.930	0.114	0.104	0.014	0.711	0.077
	Loglogistic	0.060	0.013	0.100	0.013	0.154	0.037	0.655	0.104	0.072	0.013	0.501	0.068
0.18	Blended (Spline)	0.033	0.009	0.021	0.010	0.098	0.024	0.204	0.076	0.029	0.009	0.106	0.051
	Blended (Beta)	0.039	0.012	0.020	0.001	0.121	0.037	0.033	0.023	0.026	0.007	0.090	0.027
	Weibull	0.028	0.014	0.023	0.015	0.083	0.039	0.199	0.116	0.026	0.014	0.117	0.076
	Exponential	0.111	0.011	0.169	0.011	0.283	0.031	1.137	0.087	0.129	0.011	0.855	0.056
	Lognormal	0.087	0.013	0.141	0.015	0.220	0.038	0.930	0.114	0.104	0.014	0.711	0.077
	Loglogistic	0.060	0.013	0.100	0.013	0.154	0.037	0.655	0.104	0.072	0.013	0.501	0.068
0.20	Blended (Spline)	0.044	0.009	0.031	0.010	0.131	0.023	0.287	0.075	0.040	0.009	0.157	0.053
	Blended (Beta)	0.046	0.013	0.001	0.001	0.133	0.037	0.140	0.034	0.030	0.007	0.010	0.007
	Weibull	0.028	0.014	0.023	0.015	0.083	0.039	0.199	0.116	0.026	0.014	0.117	0.076
	Exponential	0.111	0.011	0.169	0.011	0.283	0.031	1.137	0.087	0.129	0.011	0.855	0.056
	Lognormal	0.087	0.013	0.141	0.015	0.220	0.038	0.930	0.114	0.104	0.014	0.711	0.077
	Loglogistic	0.060	0.013	0.100	0.013	0.154	0.037	0.655	0.104	0.072	0.013	0.501	0.068

Supplementary Table S1D. Additional comparison with `survextrap` under 30% and 50% censoring in Scenario 1 (Weibull)

**Censoring**	**Model**	**AE (Surv)**		**AE (RMST)**		**MAE**	**FD**
		**@15y**	**@20y**	**@15y**	**@20y**	**($\pm$ SD)**	**($\pm$ SD)**
30%	Blended (Spline)	0.012	0.013	0.058	0.125	0.012 ± 0.008	0.068 ± 0.044
	Blended (Beta)	0.025	0.100	0.038	0.605	0.055 ± 0.003	0.567 ± 0.048
	**`survextrap`**	0.048	0.082	0.131	0.581	0.053 ± 0.013	0.450 ± 0.046
	Weibull	0.029	0.026	0.143	0.265	0.028 ± 0.014	0.125 ± 0.082
	Exponential	0.144	0.204	0.582	1.669	0.146 ± 0.011	1.088 ± 0.061
	Log-normal	0.116	0.173	0.461	1.391	0.119 ± 0.014	0.929 ± 0.082
	Log-logistic	0.075	0.116	0.306	0.933	0.079 ± 0.013	0.627 ± 0.075
50%	Blended (Spline)	0.040	0.029	0.192	0.328	0.036 ± 0.013	0.137 ± 0.070
	Blended (Beta)	0.008	0.100	0.228	0.429	0.074 ± 0.005	0.201 ± 0.085
	**`survextrap`**	0.019	0.078	0.139	0.362	0.055 ± 0.005	0.237 ± 0.143
	Weibull	0.104	0.092	0.508	0.958	0.100 ± 0.018	0.450 ± 0.125
	Exponential	0.227	0.294	0.974	2.521	0.228 ± 0.011	1.547 ± 0.063
	Log-normal	0.188	0.246	0.812	2.109	0.190 ± 0.015	1.297 ± 0.096
	Log-logistic	0.144	0.178	0.643	1.570	0.145 ± 0.015	0.927 ± 0.094

Notes: This supplementary table extends the main Scenario 1 comparison by adding `survextrap` under 30% and 50% censoring. Errors are evaluated over the extrapolation window $[t_{\text{obs}}, T_\star]$ with $t_{\text{obs}} = 10$ years and $T_\star = 20$ years. AE(Surv) denotes absolute survival error at 15 and 20 years; AE(RMST) denotes absolute error in restricted mean survival time from t_{obs} up to 15 and 20 years. MAE is the mean absolute survival error over the extrapolation window, and FD is defined as $|\text{AE(RMST)}_{20\text{y}} - \text{AE(RMST)}_{15\text{y}}|$.

Supplementary Table S2A. Full sensitivity results for the colorectal SEER cohorts under Low, Base, and High external-information scenarios.

**Age group**	**Scenario**	**Anchor**	**Model**	**AE (Surv)**			**AE (RMST)**			**FD**	**MAE**
				**@60m**	**@90m**	**@120m**	**@60m**	**@90m**	**@120m**		
20–59	Low	0.120	Blended (Spline)	0.026	0.003	0.035	0.959	0.907	0.933	0.027	0.026
			Blended (Beta)	0.006	0.047	0.020	0.116	1.453	1.741	0.288	0.025
			Exponential	0.055	0.085	0.089	1.012	3.731	6.476	2.744	0.066
			Weibull	0.064	0.091	0.092	1.238	4.144	6.918	2.774	0.074
			Log-normal	0.001	0.025	0.039	0.133	0.725	2.154	1.428	0.019
			Log-logistic	0.002	0.022	0.033	0.032	0.734	1.923	1.189	0.016
	Base	0.150	Blended (Spline)	0.031	0.013	0.026	1.071	1.337	0.248	1.089	0.027
			Blended (Beta)	0.004	0.066	0.049	0.092	2.090	3.484	1.393	0.038
			Exponential	0.055	0.085	0.089	1.012	3.731	6.476	2.744	0.066
			Weibull	0.064	0.091	0.092	1.238	4.144	6.918	2.774	0.074
			Log-normal	0.001	0.025	0.039	0.133	0.725	2.154	1.428	0.019
			Log-logistic	0.002	0.022	0.033	0.032	0.734	1.923	1.189	0.016
	High	0.180	Blended (Spline)	0.035	0.024	0.016	1.175	1.758	0.458	1.300	0.030
			Blended (Beta)	0.003	0.084	0.079	0.065	2.718	5.318	2.600	0.051
			Exponential	0.055	0.085	0.089	1.012	3.731	6.476	2.744	0.066
			Weibull	0.064	0.091	0.092	1.238	4.144	6.918	2.774	0.074
			Log-normal	0.001	0.025	0.039	0.133	0.725	2.154	1.428	0.019
			Log-logistic	0.002	0.022	0.033	0.032	0.734	1.923	1.189	0.016
60–69	Low	0.072	Blended (Spline)	0.026	0.007	0.022	0.898	0.984	0.276	0.708	0.023
			Blended (Beta)	0.000	0.037	0.009	0.001	1.260	1.103	0.157	0.018
			Exponential	0.051	0.061	0.057	1.055	2.938	4.595	1.657	0.053
			Weibull	0.046	0.058	0.056	0.936	2.753	4.411	1.658	0.049
			Log-normal	0.006	0.010	0.020	0.193	0.180	0.977	0.797	0.010
			Log-logistic	0.010	0.002	0.010	0.251	0.152	0.301	0.149	0.007
	Base	0.090	Blended (Spline)	0.029	0.014	0.016	0.987	1.302	0.197	1.105	0.025
			Blended (Beta)	0.001	0.052	0.027	0.015	1.738	2.260	0.522	0.028
			Exponential	0.051	0.061	0.057	1.055	2.938	4.595	1.657	0.053
			Weibull	0.046	0.058	0.056	0.936	2.753	4.411	1.658	0.049
			Log-normal	0.006	0.010	0.020	0.193	0.180	0.977	0.797	0.010
			Log-logistic	0.010	0.002	0.010	0.251	0.152	0.301	0.149	0.007
	High	0.108	Blended (Spline)	0.033	0.022	0.010	1.077	1.627	0.687	0.941	0.027
			Blended (Beta)	0.003	0.066	0.046	0.041	2.213	3.455	1.242	0.037
			Exponential	0.051	0.061	0.057	1.055	2.938	4.595	1.657	0.053
			Weibull	0.046	0.058	0.056	0.936	2.753	4.411	1.658	0.049
			Log-normal	0.006	0.010	0.020	0.193	0.180	0.977	0.797	0.010
			Log-logistic	0.010	0.002	0.010	0.251	0.152	0.301	0.149	0.007
70+	Low	0.040	Blended (Spline)	0.024	0.009	0.010	0.823	0.980	0.278	0.701	0.019
			Blended (Beta)	0.004	0.028	0.003	0.086	1.034	0.715	0.319	0.014
			Exponential	0.053	0.046	0.035	1.211	2.594	3.449	0.855	0.047
			Weibull	0.041	0.041	0.034	0.880	2.149	3.025	0.876	0.038
			Log-normal	0.003	0.009	0.011	0.025	0.345	0.707	0.362	0.007
			Log-logistic	0.006	0.002	0.001	0.137	0.172	0.173	0.001	0.003
	Base	0.050	Blended (Spline)	0.026	0.013	0.007	0.879	1.166	0.541	0.625	0.021
			Blended (Beta)	0.004	0.037	0.013	0.090	1.313	1.310	0.002	0.019
			Exponential	0.053	0.046	0.035	1.211	2.594	3.449	0.855	0.047
			Weibull	0.041	0.041	0.034	0.880	2.149	3.025	0.876	0.038
			Log-normal	0.003	0.009	0.011	0.025	0.345	0.707	0.362	0.007
			Log-logistic	0.006	0.002	0.001	0.137	0.172	0.173	0.001	0.003
	High	0.060	Blended (Spline)	0.029	0.018	0.003	0.950	1.404	0.868	0.536	0.023
			Blended (Beta)	0.006	0.046	0.022	0.115	1.639	1.953	0.313	0.025
			Exponential	0.053	0.046	0.035	1.211	2.594	3.449	0.855	0.047
			Weibull	0.041	0.041	0.034	0.880	2.149	3.025	0.876	0.038
			Log-normal	0.003	0.009	0.011	0.025	0.345	0.707	0.362	0.007
			Log-logistic	0.006	0.002	0.001	0.137	0.172	0.173	0.001	0.003
All	Low	0.072	Blended (Spline)	0.025	0.004	0.026	0.900	0.886	0.493	0.392	0.023
			Blended (Beta)	0.000	0.033	0.003	0.002	1.123	0.733	0.391	0.015
			Exponential	0.057	0.068	0.063	1.185	3.302	5.103	1.802	0.059
			Weibull	0.050	0.064	0.061	0.999	3.005	4.802	1.798	0.054
			Log-normal	0.003	0.014	0.023	0.126	0.370	1.219	0.849	0.011
			Log-logistic	0.006	0.006	0.012	0.177	0.051	0.555	0.504	0.007
	Base	0.090	Blended (Spline)	0.030	0.012	0.019	1.002	1.249	0.035	1.215	0.025
			Blended (Beta)	0.001	0.048	0.021	0.028	1.642	1.911	0.269	0.025
			Exponential	0.057	0.068	0.063	1.185	3.302	5.103	1.802	0.059
			Weibull	0.050	0.064	0.061	0.999	3.005	4.802	1.798	0.054
			Log-normal	0.003	0.014	0.023	0.126	0.370	1.219	0.849	0.011
			Log-logistic	0.006	0.006	0.012	0.177	0.051	0.555	0.504	0.007
	High	0.108	Blended (Spline)	0.033	0.019	0.013	1.085	1.548	0.496	1.051	0.027
			Blended (Beta)	0.002	0.062	0.040	0.043	2.095	3.092	0.997	0.034
			Exponential	0.057	0.068	0.063	1.185	3.302	5.103	1.802	0.059
			Weibull	0.050	0.064	0.061	0.999	3.005	4.802	1.798	0.054
			Log-normal	0.003	0.014	0.023	0.126	0.370	1.219	0.849	0.011
			Log-logistic	0.006	0.006	0.012	0.177	0.051	0.555	0.504	0.007

Notes: Anchor denotes the externally specified 120-month survival target used in the Low, Base, or High scenario. AE(Surv) = absolute survival error at 60, 90, and 120 months. AE(RMST) = absolute error in restricted mean survival time from the 36-month cutoff up to 60, 90, and 120 months. FD = $|\mathrm{AE(RMST)}_{120\mathrm{m}} - \mathrm{AE(RMST)}_{90\mathrm{m}}|$. MAE = mean absolute error over the extrapolation window. Values are rounded to three decimal places.

Supplementary Table S2B. Full sensitivity results for the melanoma SEER cohorts under Low, Base, and High external-information scenarios.

**Age group**	**Scenario**	**Anchor**	**Model**	**AE (Surv)**			**AE (RMST)**			**FD**	**MAE**
				**@60m**	**@90m**	**@120m**	**@60m**	**@90m**	**@120m**		
20–59	Low	0.200	Blended (Spline)	0.018	0.016	0.072	0.868	0.156	3.092	2.936	0.035
			Blended (Beta)	0.020	0.029	0.021	0.352	0.684	0.716	0.032	0.016
			Exponential	0.165	0.209	0.210	3.384	9.918	16.237	6.319	0.179
			Weibull	0.114	0.167	0.182	2.244	7.536	13.303	5.767	0.137
			Log-normal	0.080	0.118	0.132	1.612	5.359	9.569	4.210	0.097
			Log-logistic	0.084	0.116	0.125	1.747	5.395	9.339	3.944	0.098
	Base	0.250	Blended (Spline)	0.025	0.003	0.049	1.038	0.958	1.563	0.605	0.029
			Blended (Beta)	0.017	0.063	0.032	0.316	1.814	2.462	0.647	0.036
			Exponential	0.165	0.209	0.210	3.384	9.918	16.237	6.319	0.179
			Weibull	0.114	0.167	0.182	2.244	7.536	13.303	5.767	0.137
			Log-normal	0.080	0.118	0.132	1.612	5.359	9.569	4.210	0.097
			Log-logistic	0.084	0.116	0.125	1.747	5.395	9.339	3.944	0.098
	High	0.300	Blended (Spline)	0.032	0.019	0.029	1.186	1.609	0.250	1.360	0.031
			Blended (Beta)	0.014	0.091	0.081	0.271	2.758	5.350	2.593	0.055
			Exponential	0.165	0.209	0.210	3.384	9.918	16.237	6.319	0.179
			Weibull	0.114	0.167	0.182	2.244	7.536	13.303	5.767	0.137
			Log-normal	0.080	0.118	0.132	1.612	5.359	9.569	4.210	0.097
			Log-logistic	0.084	0.116	0.125	1.747	5.395	9.339	3.944	0.098
60–69	Low	0.160	Blended (Spline)	0.040	0.020	0.033	1.230	1.693	0.362	1.331	0.033
			Blended (Beta)	0.006	0.054	0.006	0.111	1.937	0.724	1.213	0.023
			Exponential	0.144	0.163	0.157	3.059	8.099	12.716	4.617	0.147
			Weibull	0.096	0.125	0.133	1.944	5.897	10.100	4.203	0.108
			Log-normal	0.061	0.078	0.086	1.270	3.725	6.498	2.773	0.069
			Log-logistic	0.063	0.074	0.079	1.363	3.672	6.149	2.477	0.068
	Base	0.200	Blended (Spline)	0.047	0.038	0.012	1.399	2.443	1.042	1.401	0.038
			Blended (Beta)	0.010	0.083	0.035	0.159	2.943	3.225	0.282	0.042
			Exponential	0.144	0.163	0.157	3.059	8.099	12.716	4.617	0.147
			Weibull	0.096	0.125	0.133	1.944	5.897	10.100	4.203	0.108
			Log-normal	0.061	0.078	0.086	1.270	3.725	6.498	2.773	0.069
			Log-logistic	0.063	0.074	0.079	1.363	3.672	6.149	2.477	0.068
	High	0.240	Blended (Spline)	0.053	0.054	0.007	1.536	3.094	2.306	0.788	0.047
			Blended (Beta)	0.012	0.111	0.078	0.188	3.869	5.821	1.952	0.062
			Exponential	0.144	0.163	0.157	3.059	8.099	12.716	4.617	0.147
			Weibull	0.096	0.125	0.133	1.944	5.897	10.100	4.203	0.108
			Log-normal	0.061	0.078	0.086	1.270	3.725	6.498	2.773	0.069
			Log-logistic	0.063	0.074	0.079	1.363	3.672	6.149	2.477	0.068
70+	Low	0.120	Blended (Spline)	0.041	0.025	0.003	1.246	1.863	1.150	0.713	0.030
			Blended (Beta)	0.009	0.058	0.024	0.180	2.125	2.306	0.181	0.031
			Exponential	0.117	0.119	0.092	2.547	6.177	8.465	2.288	0.110
			Weibull	0.081	0.097	0.081	1.684	4.712	6.884	2.173	0.083
			Log-normal	0.048	0.058	0.046	1.012	2.820	4.014	1.194	0.050
			Log-logistic	0.047	0.051	0.037	1.039	2.612	3.466	0.854	0.046
	Base	0.150	Blended (Spline)	0.046	0.038	0.010	1.370	2.398	2.092	0.306	0.039
			Blended (Beta)	0.011	0.080	0.053	0.210	2.864	4.092	1.228	0.045
			Exponential	0.117	0.119	0.092	2.547	6.177	8.465	2.288	0.110
			Weibull	0.081	0.097	0.081	1.684	4.712	6.884	2.173	0.083
			Log-normal	0.048	0.058	0.046	1.012	2.820	4.014	1.194	0.050
			Log-logistic	0.047	0.051	0.037	1.039	2.612	3.466	0.854	0.046
	High	0.180	Blended (Spline)	0.051	0.049	0.022	1.478	2.870	2.949	0.079	0.047
			Blended (Beta)	0.013	0.100	0.084	0.236	3.551	5.951	2.400	0.059
			Exponential	0.117	0.119	0.092	2.547	6.177	8.465	2.288	0.110
			Weibull	0.081	0.097	0.081	1.684	4.712	6.884	2.173	0.083
			Log-normal	0.048	0.058	0.046	1.012	2.820	4.014	1.194	0.050
			Log-logistic	0.047	0.051	0.037	1.039	2.612	3.466	0.854	0.046
All	Low	0.160	Blended (Spline)	0.034	0.005	0.047	1.142	1.103	1.342	0.239	0.032
			Blended (Beta)	0.000	0.040	0.015	0.017	1.388	0.001	1.387	0.016
			Exponential	0.148	0.174	0.166	3.114	8.536	13.389	4.853	0.154
			Weibull	0.101	0.139	0.145	2.037	6.429	10.905	4.475	0.116
			Log-normal	0.067	0.093	0.099	1.381	4.314	7.411	3.097	0.078
			Log-logistic	0.070	0.089	0.091	1.480	4.264	7.062	2.798	0.078
	Base	0.200	Blended (Spline)	0.041	0.022	0.027	1.303	1.828	0.001	1.827	0.034
			Blended (Beta)	0.003	0.069	0.025	0.057	2.373	2.489	0.116	0.034
			Exponential	0.148	0.174	0.166	3.114	8.536	13.389	4.853	0.154
			Weibull	0.101	0.139	0.145	2.037	6.429	10.905	4.475	0.116
			Log-normal	0.067	0.093	0.099	1.381	4.314	7.411	3.097	0.078
			Log-logistic	0.070	0.089	0.091	1.480	4.264	7.062	2.798	0.078
	High	0.240	Blended (Spline)	0.047	0.038	0.009	1.438	2.474	1.236	1.237	0.039
			Blended (Beta)	0.005	0.097	0.069	0.086	3.293	5.082	1.789	0.053
			Exponential	0.148	0.174	0.166	3.114	8.536	13.389	4.853	0.154
			Weibull	0.101	0.139	0.145	2.037	6.429	10.905	4.475	0.116
			Log-normal	0.067	0.093	0.099	1.381	4.314	7.411	3.097	0.078
			Log-logistic	0.070	0.089	0.091	1.480	4.264	7.062	2.798	0.078

Notes: Anchor denotes the externally specified 120-month survival target used in the Low, Base, or High scenario. AE(Surv) = absolute survival error at 60, 90, and 120 months. AE(RMST) = absolute error in restricted mean survival time from the 36-month cutoff up to 60, 90, and 120 months. FD = $|\mathrm{AE(RMST)}_{120\mathrm{m}} - \mathrm{AE(RMST)}_{90\mathrm{m}}|$. MAE = mean absolute error over the extrapolation window. Values are rounded to three decimal places.

Supplementary Table S2C. Full sensitivity results for the pancreatic SEER cohorts under Low, Base, and High external-information scenarios.

Age group	Scenario	Anchor	Model	AE (Surv)			AE (RMST)			FD	MAE
				@60m	@90m	@120m	@60m	@90m	@120m		
20–59	Low	0.028	Blended (Spline)	0.015	0.004	0.012	0.583	0.614	0.069	0.545	0.014
			Blended (Beta)	0.001	0.020	0.001	0.006	0.680	0.282	0.398	0.009
			Exponential	0.045	0.034	0.028	1.109	2.097	2.830	0.733	0.039
			Weibull	0.042	0.034	0.028	0.995	1.987	2.727	0.740	0.036
			Log-normal	0.028	0.027	0.025	0.650	1.466	2.165	0.699	0.027
			Log-logistic	0.020	0.019	0.018	0.459	1.046	1.580	0.534	0.019
	Base	0.035	Blended (Spline)	0.017	0.008	0.009	0.641	0.809	0.178	0.631	0.015
			Blended (Beta)	0.000	0.029	0.006	0.009	0.972	0.814	0.158	0.013
			Exponential	0.045	0.034	0.028	1.109	2.097	2.830	0.733	0.039
			Weibull	0.042	0.034	0.028	0.995	1.987	2.727	0.740	0.036
			Log-normal	0.028	0.027	0.025	0.650	1.466	2.165	0.699	0.027
			Log-logistic	0.020	0.019	0.018	0.459	1.046	1.580	0.534	0.019
	High	0.042	Blended (Spline)	0.018	0.011	0.007	0.672	0.929	0.347	0.581	0.016
			Blended (Beta)	0.000	0.035	0.013	0.012	1.168	1.251	0.083	0.017
			Exponential	0.045	0.034	0.028	1.109	2.097	2.830	0.733	0.039
			Weibull	0.042	0.034	0.028	0.995	1.987	2.727	0.740	0.036
			Log-normal	0.028	0.027	0.025	0.650	1.466	2.165	0.699	0.027
			Log-logistic	0.020	0.019	0.018	0.459	1.046	1.580	0.534	0.019
60–69	Low	0.020	Blended (Spline)	0.018	0.010	0.002	0.586	0.857	0.562	0.296	0.015
			Blended (Beta)	0.006	0.022	0.005	0.146	0.894	0.722	0.173	0.013
			Exponential	0.022	0.016	0.012	0.578	1.009	1.310	0.301	0.019
			Weibull	0.022	0.016	0.012	0.552	0.986	1.287	0.301	0.018
			Log-normal	0.015	0.013	0.011	0.368	0.756	1.055	0.299	0.014
			Log-logistic	0.008	0.008	0.007	0.184	0.421	0.632	0.210	0.008
	Base	0.025	Blended (Spline)	0.020	0.015	0.001	0.635	1.038	0.804	0.233	0.017
			Blended (Beta)	0.007	0.031	0.012	0.156	1.172	1.233	0.061	0.017
			Exponential	0.022	0.016	0.012	0.578	1.009	1.310	0.301	0.019
			Weibull	0.022	0.016	0.012	0.552	0.986	1.287	0.301	0.018
			Log-normal	0.015	0.013	0.011	0.368	0.756	1.055	0.299	0.014
			Log-logistic	0.008	0.008	0.007	0.184	0.421	0.632	0.210	0.008
	High	0.030	Blended (Spline)	0.022	0.017	0.003	0.670	1.150	0.944	0.206	0.019
			Blended (Beta)	0.008	0.035	0.016	0.166	1.339	1.537	0.198	0.020
			Exponential	0.022	0.016	0.012	0.578	1.009	1.310	0.301	0.019
			Weibull	0.022	0.016	0.012	0.552	0.986	1.287	0.301	0.018
			Log-normal	0.015	0.013	0.011	0.368	0.756	1.055	0.299	0.014
			Log-logistic	0.008	0.008	0.007	0.184	0.421	0.632	0.210	0.008
70+	Low	0.004	Blended (Spline)	0.007	0.003	0.002	0.285	0.342	0.171	0.172	0.006
			Blended (Beta)	0.001	0.008	0.001	0.047	0.302	0.124	0.178	0.004
			Exponential	0.014	0.008	0.006	0.368	0.582	0.710	0.128	0.011
			Weibull	0.014	0.008	0.006	0.357	0.572	0.700	0.128	0.011
			Log-normal	0.011	0.007	0.005	0.263	0.463	0.592	0.129	0.009
			Log-logistic	0.005	0.004	0.003	0.118	0.221	0.298	0.077	0.004
	Base	0.005	Blended (Spline)	0.008	0.004	0.001	0.320	0.420	0.252	0.168	0.007
			Blended (Beta)	0.002	0.010	0.001	0.057	0.399	0.244	0.155	0.005
			Exponential	0.014	0.008	0.006	0.368	0.582	0.710	0.128	0.011
			Weibull	0.014	0.008	0.006	0.357	0.572	0.700	0.128	0.011
			Log-normal	0.011	0.007	0.005	0.263	0.463	0.592	0.129	0.009
			Log-logistic	0.005	0.004	0.003	0.118	0.221	0.298	0.077	0.004
	High	0.006	Blended (Spline)	0.009	0.004	0.001	0.331	0.432	0.274	0.158	0.008
			Blended (Beta)	0.002	0.010	0.001	0.057	0.408	0.285	0.123	0.005
			Exponential	0.014	0.008	0.006	0.368	0.582	0.710	0.128	0.011
			Weibull	0.014	0.008	0.006	0.357	0.572	0.700	0.128	0.011
			Log-normal	0.011	0.007	0.005	0.263	0.463	0.592	0.129	0.009
			Log-logistic	0.005	0.004	0.003	0.118	0.221	0.298	0.077	0.004
All	Low	0.016	Blended (Spline)	0.015	0.007	0.004	0.538	0.693	0.320	0.372	0.013
			Blended (Beta)	0.003	0.019	0.003	0.088	0.725	0.535	0.190	0.010
			Exponential	0.026	0.019	0.015	0.686	1.212	1.585	0.373	0.023
			Weibull	0.026	0.019	0.015	0.644	1.173	1.548	0.375	0.022
			Log-normal	0.019	0.016	0.014	0.445	0.915	1.284	0.369	0.017
			Log-logistic	0.011	0.010	0.010	0.261	0.569	0.838	0.270	0.010
	Base	0.020	Blended (Spline)	0.017	0.009	0.003	0.575	0.801	0.437	0.364	0.014
			Blended (Beta)	0.004	0.023	0.006	0.098	0.879	0.755	0.123	0.012
			Exponential	0.026	0.019	0.015	0.686	1.212	1.585	0.373	0.023
			Weibull	0.026	0.019	0.015	0.644	1.173	1.548	0.375	0.022
			Log-normal	0.019	0.016	0.014	0.445	0.915	1.284	0.369	0.017
			Log-logistic	0.011	0.010	0.010	0.261	0.569	0.838	0.270	0.010
	High	0.024	Blended (Spline)	0.017	0.010	0.003	0.580	0.832	0.486	0.346	0.014
			Blended (Beta)	0.004	0.026	0.008	0.094	0.943	0.906	0.037	0.014
			Exponential	0.026	0.019	0.015	0.686	1.212	1.585	0.373	0.023
			Weibull	0.026	0.019	0.015	0.644	1.173	1.548	0.375	0.022
			Log-normal	0.019	0.016	0.014	0.445	0.915	1.284	0.369	0.017
			Log-logistic	0.011	0.010	0.010	0.261	0.569	0.838	0.270	0.010

Notes: Anchor denotes the externally specified 120-month survival target used in the Low, Base, or High scenario. AE(Surv) = absolute survival error at 60, 90, and 120 months. AE(RMST) = absolute error in restricted mean survival time from the 36-month cutoff up to 60, 90, and 120 months. FD = $|\text{AE(RMST)}_{120\text{m}} - \text{AE(RMST)}_{90\text{m}}|$. MAE = mean absolute error over the extrapolation window. Values are rounded to three decimal places.

References

[1] Jackson C. *flexsurv*: a platform for parametric survival modeling in r. *J Stat Softw* 2016; 70: 1–33.

[2] Gompertz B. On the nature of the function expressive of the law of human mortality, and on a new mode of determining the value of life contingencies. In a letter to Francis Baily, esq. frs &c. by benjamin gompertz, esq. fr s. In: *Abstracts of the papers printed in the Philosophical Transactions of the Royal Society of London*, 1833, 2, pp.252–253. The Royal Society London.

[3] Lawless JF. *Statistical Models and Methods for Lifetime Data*. 2nd ed. Hoboken, NJ: John Wiley & Sons, 2011.

[4] Klein JP and Moeschberger ML. *Survival Analysis: Techniques for Censored and Truncated Data*. 2nd ed. New York, NY: Springer-Verlag, 2003.
